# Supplementary material for: Effects and cost-effectiveness of postoperative oral analgesics for additional postoperative pain relief in children and adolescents undergoing dental treatment: Health technology assessment including a systematic review
Source: PLoS One. 2019 Dec 31;14(12):e0227027. doi: 10.1371/journal.pone.0227027 (PMC6938383; doi:10.1371/journal.pone.0227027)
Supplement: S1 File — (DOCX) [file pone.0227027.s002.docx]

**S1. Search strategies**

**PubMed via NLM, 20^th^ November 2018**

| 1. Population | Children and Young adults (In title and abstract) |  |
| --- | --- | --- |
|  | child[Title/Abstract] OR children[Title/Abstract] OR infant[Title/Abstract] OR infants[Title/Abstract] OR adolescent[Title/Abstract] OR adolescents[Title/Abstract] OR adolescence[Title/Abstract] OR Minor[Title/Abstract] OR Minors[Title/Abstract] OR teenager[Title/Abstract] OR teenagers[Title/Abstract] OR teen[Title/Abstract] OR teens[Title/Abstract] AND Young[Title/Abstract] OR Youth[Title/Abstract] OR Youths[Title/Abstract] OR student[Title/Abstract] OR students[Title/Abstract] OR offspring[Title/Abstract] OR puberty[Title/Abstract] OR juvenile[Title/Abstract] OR juveniles[Title/Abstract] OR toddler[Title/Abstract] OR toddlers[Title/Abstract] OR School age[Title/Abstract] OR boys[Title/Abstract] OR girls[Title/Abstract] OR pediatric[Title/Abstract] OR pediatrics[Title/Abstract] OR paediatrics[Title/Abstract] OR paediatric[Title/Abstract] | 928 576 |
| 2. Population | Children and Young adults (In Medical Subject Headings) |  |
|  | "Child"[Mesh] OR "Infant"[Mesh] OR "Adolescent"[Mesh] OR "Minors"[Mesh] OR "Students"[Mesh] OR "Puberty"[Mesh] OR "Pediatrics"[Mesh] | 3 446 133 |
| 3. | 1 OR 2 | 3 778 924 |
|  |  |  |
| 4.Intervention | Dental care (In title and abstract) |  |
|  | dental care[Title/Abstract] OR dental care for children[Title/Abstract] OR dentistry for children[Title/Abstract] OR pediatric dentistry[Title/Abstract] OR filling therapy[Title/Abstract] OR dental restoration[Title/Abstract] OR dental treatment[Title/Abstract] OR teeth extraction[Title/Abstract] OR teeth extractions[Title/Abstract] OR tooth extractions[Title/Abstract] OR tooth extraction[Title/Abstract] OR pulp capping[Title/Abstract] OR pulp therapy[Title/Abstract] OR pulpotomy[Title/Abstract] OR pulpotomies[Title/Abstract] OR pulpectomy[Title/Abstract] OR pulpectomies[Title/Abstract] OR endodontic[Title/Abstract] OR endodontics[Title/Abstract] OR endodontology[Title/Abstract]) OR root canal[Title/Abstract] OR oral surgery[Title/Abstract] OR maxillofacial surgery[Title/Abstract] OR minor oral surgery[Title/Abstract] OR tooth removal[Title/Abstract] OR teeth removal[Title/Abstract] OR caries[Title/Abstract] OR carious[Title/Abstract] OR tooth decay[Title/Abstract] OR teeth decay[Title/Abstract] OR tooth replantation[Title/Abstract] OR replantation of teeth[Title/Abstract] OR tooth replantations[Title/Abstract] OR tooth reimplantations[Title/Abstract] OR tooth reimplantation[Title/Abstract] | 101 882 |
| 5.Intervention | Dental care (In Medical Subject Headings) |  |
|  | "Pediatric Dentistry"[Mesh] OR "Dental Care for Children"[Mesh] OR "Dental Care"[Mesh] OR "Dental Restoration, Temporary"[Mesh] OR "Dental Restoration, Permanent"[Mesh] OR "Tooth Extraction"[Mesh] OR "Dental Pulp Capping"[Mesh] OR "Pulpotomy"[Mesh] OR "Pulpectomy"[Mesh] OR "Endodontics"[Mesh] OR "Root Canal Therapy"[Mesh] OR "Surgery, Oral"[Mesh] OR "Oral Surgical Procedures"[Mesh] OR "Root Caries"[Mesh] OR "Tooth Replantation"[Mesh] | 145 300 |
| 6. | 4 OR 5 | 205 071 |
|  |  |  |
| 7.Intervention | Analgesia (In title and abstract) |  |
|  | aspirin like agents[Title/Abstract] OR aspirin like agent[Title/Abstract] OR aspirinlike agent[Title/Abstract] OR aspirinlike agents[Title/Abstract] OR aspirin-like agents[Title/Abstract] OR aspirin-like agent[Title/Abstract] OR acetysal[Title/Abstract] OR zorprin[Title/Abstract] OR solupsan[Title/Abstract] OR solprin[Title/Abstract] OR polopiryna[Title/Abstract] OR polopirin[Title/Abstract] OR micristin[Title/Abstract] OR magnecyl[Title/Abstract] OR endosprin[Title/Abstract] OR ecotrin[Title/Abstract] OR easprin[Title/Abstract] OR dispril[Title/Abstract] OR colfarit[Title/Abstract] OR aloxiprimum[Title/Abstract] OR acylpyrin[Title/Abstract] OR acetylsalicylic acid[Title/Abstract] OR aspirin[Title/Abstract] OR aleve[Title/Abstract] OR synflex[Title/Abstract] OR proxen[Title/Abstract] OR naprosyn[Title/Abstract] OR naprosin[Title/Abstract] OR anaprox[Title/Abstract] OR MNPA[Title/Abstract] OR methoxypropiocin[Title/Abstract] OR naproxen[Title/Abstract] OR Voltaren[Title/Abstract] OR orthophen[Title/Abstract] OR ortofen[Title/Abstract] OR orthofen[Title/Abstract] OR novapirina[Title/Abstract] OR voltarol[Title/Abstract] OR feloran[Title/Abstract] OR diclonate[Title/Abstract] OR dichlofenal[Title/Abstract] OR diclofenac[Title/Abstract] OR diclophenac[Title/Abstract] OR algotropyl[Title/Abstract] OR acamol[Title/Abstract] OR panadol[Title/Abstract] OR datril[Title/Abstract] OR anacin[Title/Abstract] OR tylenol[Title/Abstract] OR acetaco[Title/Abstract] OR acephen[Title/Abstract] OR analgesias[Title/Abstract] OR analgesia[Title/Abstract] OR analgesics[Title/Abstract] OR analgesic[Title/Abstract] OR NSAIDs[Title/Abstract] OR NSAID[Title/Abstract] OR analgesi[Title/Abstract] OR anti-inflammatory[Title/Abstract] OR antiinflammatory[Title/Abstract] OR anti inflammatory[Title/Abstract] OR anti inflammator[Title/Abstract] OR anti-inflammator[Title/Abstract] OR anti-inflammatories[Title/Abstract] OR anti inflammatories[Title/Abstract] OR antiinflammatories[Title/Abstract] OR ibuprofen[Title/Abstract] OR brufen[Title/Abstract] OR ibumetin[Title/Abstract] OR motrin[Title/Abstract] OR nuprin[Title/Abstract] OR rufen[Title/Abstract] OR nuprin[Title/Abstract] OR rufen[Title/Abstract] OR salprofen[Title/Abstract] OR acetaminophen[Title/Abstract] OR paracetamol[Title/Abstract] OR hydroxyacetanilide[Title/Abstract] OR acetamidophenol[Title/Abstract] | 339 108 |
| 8.Intervention | Analgesia (In Medical Subject Headings) |  |
|  | "Aspirin"[Mesh] OR "Naproxen"[Mesh] OR "Diclofenac"[Mesh] OR "Acetaminophen"[Mesh] OR "Ibuprofen"[Mesh] OR "Anti-Inflammatory Agents, Non-Steroidal"[Mesh] OR "Anti-Inflammatory Agents"[Mesh] OR "Analgesics"[Mesh] OR "Analgesia"[Mesh] | 325 001 |
| 9. | 7 OR 8 | 497 799 |
|  |  |  |
|  | 3 AND 6 AND 9 | 1 216 |
|  |  |  |

**Cochrane via Wiley, 20^th^ November 2018**

| 1.Population | Children and Young adults (in title, abstract and keywords) |  |
| --- | --- | --- |
|  | ti,ab,kw (Word variations have been searched) child OR children OR infant OR infants OR adolescent OR adolescence OR minor OR teenager OR teen OR young OR youth OR student OR offspring OR puberty OR juvenile OR toddler OR school age OR boys OR girls OR pediatric OR paediatric OR “dental care for children” OR “dentistry for children” | 293 739 |
| 2. Population | Children and Young adults (In Medical Subject Headings) |  |
|  | Child[MeSH] OR Infant[MeSH] OR Adolescent[MeSH] OR Minors[MeSH] OR Students[MeSH] OR Puberty[MeSH] OR Pediatrics[MeSH] OR Pediatric Dentistry[MeSH] OR Dental Care for Children[MeSH] | 112 418 |
| 3. | 1 OR 2 | 293 751 |
|  |  |  |
| 4.Intervention | Dental Care (In title, abstract and keywords) |  |
|  | ti,ab,kw (Word variations have been searched) dental care OR dental restoration OR dental treatment OR teeth extraction OR tooth extraction OR pulp capping OR pulp therapy OR pulpotomy OR pulpotomies OR pulpectomy OR pulpectomies OR endodontic OR endodontology OR root canal OR oral surgery OR maxillofacial surgery OR minor oral surgery OR tooth removal OR teeth removal OR caries OR carious OR tooth decay OR teeth decay OR tooth replantation OR replantation of teeth OR tooth reinplantation | 29 474 |
| 5.Intervention | Dental Care (In Medical Subject Headings) |  |
|  | Dental Care[MeSH] OR Dental Restoration, Permanent[MeSH] OR Dental Restoration, Temporary[MeSH] OR Tooth Extraction[MeSH] OR Dental Pulp Capping[MeSH] OR Pulpotomy[MeSH] OR Pulpectomy[MeSH] OR Endodontics[MeSH] OR Root Canal Surgery[MeSH] OR Surgery, Oral[MeSH] OR Oral Surgical Procedures[MeSH] OR Root Caries[MeSH] OR Tooth Reinplantation[MeSH] | 7 325 |
| 6. | 3 OR 4 | 30 403 |
|  |  |  |
| 7.Intervention | Analgesia (In title, abstract and keywords) |  |
|  | ti,ab,kw (Word variations have been searched) analgesi OR analgesia OR analgesic OR NSAID OR anti-inflammatory agent OR anti inflammatory agent OR antiinflammatory agent OR anti-inflammatory OR anti inflammatory OR antiinflammatories OR anti-inflammatories OR anti inflammatories OR anti-inflammator OR anti inflammatory OR ibuprofen OR brufen OR ibumetin OR motrin OR nuprin OR rufen OR salprofen OR acetaminophen OR paracetamol OR hydroxyacetanilide OR acetamidophenol OR acephen OR acetaco OR Tylenol OR anacin OR datril OR Panadol OR acamol OR algotrypyl OR diclofenac OR diclophenac OR dicrofenac OR dichlofenal OR diclonate OR feloran OR voltarol OR novapirina OR orthofen OR ortofen OR othophen OR voltaren OR naproxen OR methoxypropiocin OR MNPA OR anaprox OR naprosin OR naprosyn OR proxen OR synflex OR aleve OR aspirin OR acetylsalicylic acid OR acylpyrin OR aloxiprimum OR colfarit OR dispril OR easprin OR ecotrin OR endosprin OR magnecyl OR micristin OR polopirin OR polopiryna OR solprin OR solupsan OR zorprin OR acetysal OR aspirinlike agent OR aspirin-like agent OR aspirin like agent | 79 301 |
| 8.Intervention | Analgesia (In Medical Subject Headings) |  |
|  | Analgesia[MeSH] OR Analgesics[MeSH] OR Anti-Inflammatory Agents[MeSH] OR Anti-Inflammatory Agents, Non-Steriodal[MeSH] OR Ibuprofen[MeSH] OR Acetaminophen[MeSH] OR Diclofenac[MeSH] OR Naproxen[MeSH] OR Aspirin[MeSH] | 35 551 |
| 9. | 7 OR 8 | 85 061 |
|  |  |  |
|  | 3 AND 6 AND 9 | 1 760 |

**Scopus, 21^th^ November 2018**

| 1.Population | Children and Young adults (in title, abstract and keywords) |  |
| --- | --- | --- |
|  | (TITLE-ABS-KEY) child OR children OR infant OR infants OR adolescent OR adolescents OR adolescence OR minor OR minors OR teenager OR teenagers OR teen OR teens OR young OR youth OR youths OR student OR students OR offspring OR puberty OR juvenile OR juveniles OR toddler OR toddlers OR “school age” OR boys OR girls OR pediatric OR pediatrics OR paediatric OR paediatrics | 6 976 098 |
| 2.Intervention | Dental Care (In title, abstract and keywords) |  |
|  | (TITLE-ABS-KEY) “dental care” OR “dental care for children” OR “dental restoration” OR “dental treatment” OR “dentistry for children” OR “pediatric dentistry” OR “teeth extraction” OR “teeth extractions” OR “tooth extraction” OR “tooth extractions” OR “pulp capping” OR “pulp therapy” OR pulpotomy OR pulpotomies OR pulpectomy OR pulpectomies OR endodontic OR endodontics OR endodontology OR “root canal” OR “oral surgery” OR “maxillofacial surgery” OR “minor oral surgery” OR “tooth removal” OR “teeth removal” OR caries OR carious OR “tooth decay” OR “teeth decay” OR “tooth replantation” OR “replantation of teeth” OR “teeth replantation” OR “tooth reimplantation” OR “tooth reimplantations” OR “filling theraphy” | 244 113 |
| 3.Intervention | Analgesia (In title, abstract and keywords) |  |
|  | (TITLE-ABS-KEY) analgesi OR analgesia OR analgesias OR analgesic OR analgesics OR NSAID OR NSAIDs OR “anti-inflammatory” OR “anti inflammatory” OR “antiinflammatory” OR antiinflammatories OR “anti-inflammatories” OR “anti inflammatories” OR “anti-inflammator” OR “anti inflammatory” OR ibuprofen OR brufen OR ibumetin OR motrin OR nuprin OR rufen OR salprofen OR acetaminophen OR paracetamol OR hydroxyacetanilide OR acetamidophenol OR acephen OR acetaco OR Tylenol OR anacin OR datril OR Panadol OR acamol OR algotrypyl OR diclofenac OR diclophenac OR dichlofenal OR diclonate OR feloran OR voltarol OR novapirina OR orthofen OR ortofen OR othophen OR voltaren OR naproxen OR methoxypropiocin OR MNPA OR anaprox OR naprosin OR naprosyn OR proxen OR synflex OR aleve OR aspirin OR “acetylsalicylic acid” OR acylpyrin OR aloxiprimum OR colfarit OR dispril OR easprin OR ecotrin OR endosprin OR magnecyl OR micristin OR polopirin OR polopiryna OR solprin OR solupsan OR zorprin OR acetysal OR “aspirinlike agent” OR “aspirin-like agent” OR “aspirin like agent” | 832 784 |
|  |  |  |
|  | 1 AND 2 AND 3 | 1 972 |

**Cinahl via Ebsco, 23^rd^ November 2018**

| 1.Population | Children and Young adults (in title and abstract) |  |
| --- | --- | --- |
|  | AB ( child OR children OR infant OR infants OR adolescent OR adolescents OR adolescence OR minor OR minors OR teenager OR teenagers OR teen OR teens OR young OR youth OR youths OR student OR students OR offspring OR puberty OR juvenile OR juveniles OR toddler OR toddlers OR “school age” OR boys OR girls OR pediatric OR pediatrics OR paediatric OR paediatrics ) OR TI ( child OR children OR infant OR infants OR adolescent OR adolescents OR adolescence OR minor OR minors OR teenager OR teenagers OR teen OR teens OR young OR youth OR youths OR student OR students OR offspring OR puberty OR juvenile OR juveniles OR toddler OR toddlers OR “school age” OR boys OR girls OR pediatric OR pediatrics OR paediatric OR paediatrics ) | 441 435 |
| 2. Population | Children and Young adults (in Cinahl Headings) |  |
|  | (MH “adolescence+”) OR (MH “Child+”) OR (MH "Minors (Legal)") OR (MH "Infant+") OR (MH "Young Adult") OR (MH "Students+") OR (MH "Puberty+") | 974 565 |
| 3. | 1 OR 2 | 1 210 794 |
|  |  |  |
| 4.Intervention | Dental Care (in title and abstract) |  |
|  | TI ( “dental care” OR “dental care for children” OR “dental restoration” OR “dental treatment” OR “dentistry for children” OR “pediatric dentistry” OR “teeth extraction” OR “teeth extractions” OR “tooth extraction” OR “tooth extractions” OR “pulp capping” OR “pulp therapy” OR pulpotomy OR pulpotomies OR pulpectomy OR pulpectomies OR endodontic OR endodontics OR endodontology OR “root canal” OR “oral surgery” OR “maxillofacial surgery” OR “minor oral surgery” OR “tooth removal” OR “teeth removal” OR caries OR carious OR “tooth decay” OR “teeth decay” OR “tooth replantation” OR “replantation of teeth” OR “teeth replantation” OR “tooth reimplantation” OR “tooth reimplantations” OR “filling theraphy” ) OR AB ( “dental care” OR “dental care for children” OR “dental restoration” OR “dental treatment” OR “dentistry for children” OR “pediatric dentistry” OR “teeth extraction” OR “teeth extractions” OR “tooth extraction” OR “tooth extractions” OR “pulp capping” OR “pulp therapy” OR pulpotomy OR pulpotomies OR pulpectomy OR pulpectomies OR endodontic OR endodontics OR endodontology OR “root canal” OR “oral surgery” OR “maxillofacial surgery” OR “minor oral surgery” OR “tooth removal” OR “teeth removal” OR caries OR carious OR “tooth decay” OR “teeth decay” OR “tooth replantation” OR “replantation of teeth” OR “teeth replantation” OR “tooth reimplantation” OR “tooth reimplantations” OR “filling theraphy” ) | 19 692 |
| 5.Intervention | Dental Care (in Cinahl Headings) |  |
|  | (MH "Dental Care+") OR (MH "Dental Care for Children") OR (MH "Dental Restoration, Temporary") OR (MH "Dental Restoration, Permanent+") OR (MH "Tooth Extraction") OR (MH "Pediatric Dentistry") OR (MH "Pulpotomy") OR (MH "Pulpectomy") OR (MH "Endodontics+") OR (MH "Root Canal Therapy") OR (MH "Surgery, Oral+") OR (MH "Dental Caries") OR (MH "Tooth Replantation") | 47 423 |
| 6. | 4 OR 5 | 53 965 |
|  |  |  |
| 7.intervention | Analgesia (In title and abstract) |  |
|  | TI ( analgesi OR analgesia OR analgesias OR analgesic OR analgesics OR NSAID OR NSAIDs OR “anti-inflammatory” OR “anti inflammatory” OR “antiinflammatory” OR antiinflammatories OR “anti-inflammatories” OR “anti inflammatories” OR “anti-inflammator” OR “anti inflammatory” OR ibuprofen OR brufen OR ibumetin OR motrin OR nuprin OR rufen OR salprofen OR acetaminophen OR paracetamol OR hydroxyacetanilide OR acetamidophenol OR acephen OR acetaco OR Tylenol OR anacin OR datril OR Panadol OR acamol OR algotrypyl OR diclofenac OR diclophenac OR dichlofenal OR diclonate OR feloran OR voltarol OR novapirina OR orthofen OR ortofen OR othophen OR voltaren OR naproxen OR methoxypropiocin OR MNPA OR anaprox OR naprosin OR naprosyn OR proxen OR synflex OR aleve OR aspirin OR “acetylsalicylic acid” OR acylpyrin OR aloxiprimum OR colfarit OR dispril OR easprin OR ecotrin OR endosprin OR magnecyl OR micristin OR polopirin OR polopiryna OR solprin OR solupsan OR zorprin OR acetysal OR “aspirinlike agent” OR “aspirin-like agent” OR “aspirin like agent” ) OR AB ( analgesi OR analgesia OR analgesias OR analgesic OR analgesics OR NSAID OR NSAIDs OR “anti-inflammatory” OR “anti inflammatory” OR “antiinflammatory” OR antiinflammatories OR “anti-inflammatories” OR “anti inflammatories” OR “anti-inflammator” OR “anti inflammatory” OR ibuprofen OR brufen OR ibumetin OR motrin OR nuprin OR rufen OR salprofen OR acetaminophen OR paracetamol OR hydroxyacetanilide OR acetamidophenol OR acephen OR acetaco OR Tylenol OR anacin OR datril OR Panadol OR acamol OR algotrypyl OR diclofenac OR diclophenac OR dichlofenal OR diclonate OR feloran OR voltarol OR novapirina OR orthofen OR ortofen OR othophen OR voltaren OR naproxen OR methoxypropiocin OR MNPA OR anaprox OR naprosin OR naprosyn OR proxen OR synflex OR aleve OR aspirin OR “acetylsalicylic acid” OR acylpyrin OR aloxiprimum OR colfarit OR dispril OR easprin OR ecotrin OR endosprin OR magnecyl OR micristin OR polopirin OR polopiryna OR solprin OR solupsan OR zorprin OR acetysal OR “aspirinlike agent” OR “aspirin-like agent” OR “aspirin like agent” ) | 58 849 |
| 8.Intervention | Analgesia (In Cinahl Headings) |  |
|  | (MH “Antiinflammatory Agents, Non Steriodal+”) OR (MH “Aspisrin”) OR (MH “Naproxen”) OR (MH “Diclofenac”) OR (MH”Acetaminophen”) OR (MH ”Ibuprofen”) OR (MH “Antiinflammatory Agents+”) OR (MH “Analgesia+”) OR (MH "Analgesics+") | 105 715 |
| 9. | 7 OR 8 | 133 178 |
|  |  |  |
|  | 3 AND 6 AND 9 | 469 |
|  |  |  |

**Embase, 23^rd^ November 2018**

| 1.Population | Children and Young adults (in title, abstract and keywords) |  |
| --- | --- | --- |
|  | Ti,ab,kw ‘child’ OR ‘children’ OR ‘infant’ OR ‘infants’ OR ‘adolescents’ OR ‘adolescence’ OR ‘minor’ OR ‘minors’ OR ‘teenager’ OR ‘teenagers’ OR ‘teen’ OR ‘teens’ OR ‘young’ OR ‘youth’ OR ‘youths’ OR ‘student’ OR ‘students’ OR ‘offspring’ OR ‘puberty’ OR ‘juvenile’ OR ‘juveniles’ OR ‘toddler’ OR ‘toddlers’ OR ‘school age’ OR ‘boys’ OR ‘girls’ OR ‘pediatric’ OR ‘pediatrics’ OR ‘paediatric’ OR ‘paediatrics’ | 3 210 057 |
| 2.Population | Children and Young adults (in Emtree) |  |
|  | ‘Child’/exp OR ‘newborn’/exp OR ‘adolescent’/exp OR ‘minor (person)’/exp OR ‘student’/exp OR ‘puberty’/exp OR ‘pediatrics’/exp | 4 441 292 |
| 3. | 1 OR 2 | 5 400 300 |
|  |  |  |
| 4.intervention | Dental Care (In title, abstract and keywords) |  |
|  | Ti,ab,kw ‘dental care’ OR ‘dental care for children’ OR ‘dentistry for children’ OR ‘pediatric dentistry’ OR ‘filling theraphy’ OR ‘dental restoration’ OR ‘dental treatment’ OR ‘teeth extration’ OR ‘teeth extractions’ OR ‘tooth extraction’ OR ‘tooth extractions’ OR ‘pulp capping’ OR ‘pulp theraphy’ OR ‘polpotomy’ OR ‘pulpotomies’ OR ‘pulpectomy’ OR ‘pulpectomies’ OR ‘endodontic’ OR ‘endodontics’ OR ‘endodontology’ OR ‘root canal’ OR ‘oral surgery’ OR ‘maxillofacial surgery’ OR ‘minor oral surgery’ OR ‘tooth removal’ OR ‘teeth removal’ OR ‘caries’ OR ‘carious’ OR ‘tooth decay’ OR ‘teeth decay’ OR ‘tooth replantation’ OR ‘reinplantation of teeth’ OR ‘tooth replantations’ OR ‘tooth reimplantations’ OR ‘tooth reimplantation’ | 103 047 |
| 5.Intervention | Dental Care (In Emtree) |  |
|  | ‘dental restoration’/exp OR ‘tooth extraction’/exp OR ‘dental pulp capping’/exp OR ‘pulpotomy’/exp OR ‘pulpectomy’/exp OR ‘endodontics’/exp OR ‘oral surgery’/exp OR ‘dental caries’/exp OR ‘tooth replantation’/exp | 237 978 |
| 6. | 4 OR 5 | 265 895 |
|  |  |  |
| 7.Intervention | Analgesia (In title, abstract and keywords) |  |
|  | Ti,ab,kw: ‘aspirin like agents’ OR ‘aspirin like agent’ OR ‘aspirinlike agent’ OR ‘aspirinlike agents’ OR ‘aspirin-like agents’ ‘aspirin-like agent’ OR ‘acetysal’ OR ‘zorpin’ OR ‘solupsan’ OR ‘solprin’ OR ‘polopirin’ OR ‘polopiryna’ OR ‘micristin’ OR ‘magnecyl’ OR ‘endosprin’ OR ‘ecotrin’ OR ‘easprin’ OR ‘dispril’ OR ‘colfarit’ OR ‘aloxiprimum’ OR ‘acylpyrin’ OR ‘acetylsalicylic acid’ OR ‘aspirin’ OR ‘aleve’ OR ‘synflex’ OR ‘proxen’ OR ‘naprosyn’ OR ‘naprosin’ OR ‘anaprox’ OR ‘mnpa’ OR ‘methoxypropiocin’ OR ‘naproxen’ OR ‘voltaren’ OR ‘orthophen’ OR ‘ortofen’ OR ‘orthofen’ OR ‘novapirina’ OR ‘voltarol’ OR ‘feloran’ OR ‘diclonate’ OR ‘dichlofenal’ OR ‘diclofenac’ OR ‘ dichlophenac’ OR ‘algotrophyl’ OR ‘acamol’ OR ‘panadol’ OR ‘datril’ OR ‘anacin’ OR ‘tylenol’ OR ‘acetaco’ OR ‘acephen’ OR ‘analgesias’ OR ‘analgesia’ OR ‘analgesics’ OR ‘analgesic’ OR ‘nsaids’ OR ‘nsaid’ OR ‘analgesi’ OR ‘anti-inflammatory’ OR ‘anti inflammatory’ OR ‘anti-inflammator’ OR ‘anti-inflammatories’ OR ‘anti inflammatories’ OR ‘antiinflammatories’ OR ‘ibuprofen’ OR ‘ brufen’ OR ‘ ibumetin’ OR ‘motrin’ OR ‘nuprin’ OR ‘rufen’ OR ‘salprofen’ OR ‘acetaminophen’ OR ‘paracetamol’ OR ‘hydroxyacetanilide’ OR ‘acetamidophenol’ | 470 671 |
| 8.Intervention | Analgesia (In Emtree) |  |
|  | ‘analgesia’/exp OR ‘analgesic agent’/exp OR ‘antiinflammatory agent’/exp OR ‘ibuprofen’/exp OR ‘paracetamol’/exp OR ‘diclofenac’/exp OR ‘naproxen’/exp OR ‘acetylsalicylic acid’/exp | 2 324 963 |
| 9. | 7 OR 8 | 2 440 141 |
|  |  |  |
|  | 3 AND 6 AND 9 | 2586 |
