# Supplementary material for: Effects and cost-effectiveness of postoperative oral analgesics for additional postoperative pain relief in children and adolescents undergoing dental treatment: Health technology assessment including a systematic review
Source: PLoS One. 2019 Dec 31;14(12):e0227027. doi: 10.1371/journal.pone.0227027 (PMC6938383; doi:10.1371/journal.pone.0227027)
Supplement: S2 File — List of excluded full text papers. (DOCX) [file pone.0227027.s003.docx]

**S2. Characteristics of excluded studies**

**List of excluded full text papers**

| **Reference number** | **Name of first author, year** | **Reason for exclusion** |
| --- | --- | --- |
| 1 | Akbulut, 2014 | No data for 0-19 yrs |
| 2 | Alpaslan, 1997 | No data for 0-19 yrs. |
| 3 | Arribas, 2003 | Narrative |
| 4 | Arslan, 2011 | No data for 0-19 yrs |
| 5 | Atkinson, 2015 | No data for 0-19 yrs |
| 6 | Averbuch, 2001 | No data for 0-19 yrs |
| 7 | Bailey, 1993 | No data for 0-19 yrs |
| 8 | Best, 2017 | No data for 0-19 yrs |
| 9 | Björnsson, 2003 | No data for 0-19 yrs |
| 10 | Björnsson, 2003 | No data for 0-19 yrs |
| 11 | Björnsson, 2011 | Adult patients |
| 12 | Bocanegra, 2005 | No data for 0-19 yrs |
| 13 | Boerlin, 1986 | No data for 0-19 yrs |
| 14 | Bracco, 2008 | No data för 0-19 |
| 15 | Brain, 2015 | No data for 0-19 yrs |
| 16 | Breivik, 1998 | No data for 0-19 yrs |
| 17 | Chang, 2001 | Use of narcotic analgesic |
| 18 | Chang, 2002 | No data for 0-19 yrs |
| 19 | Chang, 2004 | Use of narcotic analgesic |
| 20 | Chang, 2005 | Use of narcotic analgesic |
| 21 | Chopra, 2009 | No data for 0-19 yrs |
| 22 | Christensen, 2017 | No data for 0-19 yrs |
| 23 | Cochrane, 2002 | Review, narrative |
| 24 | Cooper, 1982 | No data for 0-19 yrs |
| 25 | Cooper, 1984 | No data for 0-19 yrs |
| 26 | Cooper, 1989 | Only adults |
| 27 | Coulthard, 2000 | No data for 0-19 yrs |
| 28 | Coulthard, 2001 | No data for 0-19 yrs |
| 29 | Daniels, 2006 | No data for 0-19 yrs |
| 30 | Daniels, 2009 | No data for 0-19 yrs |
| 31 | Daniels, 2011 | No data for 0-19 yrs |
| 32 | Daniels, 2011 | No data for 0-19 yrs |
| 33 | Desjardins, 1983 | No data for 0-19 yrs |
| 34 | Desjardins, 1984 | No data for 0-19 yrs |
| 35 | Dietrich, 2017 | Adult patients |
| 36 | Dionne, 1984 | No data for 0-19 yrs |
| 37 | Dodd, 2002 | Case report |
| 38 | Doyle, 2002 | No data for 0-19 yrs |
| 39 | Edmondson, 1983 | No data for 0-19 yrs |
| 40 | El Batawi, 2015 | Treatment under general anesthesia |
| 41 | Elhakim, 1993 | Treatment under general anesthesia |
| 42 | Eroglu, 2014 | Only adults |
| 43 | Estellar-Martinez, 2004 | No data for 0-19 yrs |
| 44 | Forbes, 1980 | No data for 0-19 yrs |
| 45 | Forbes, 1984 | No data for 0-19 yrs |
| 46 | Forbes, 1986 | No data for 0-19 yrs |
| 47 | Forbes, 1989 | No data for 0-19 yrs |
| 48 | Forbes, 1991 | No data for 0-19 yrs |
| 49 | Forbes, 1992 | No data for 0-19 yrs |
| 50 | Frame, 1986 | No data for 0-19 yrs |
| 51 | Frame, 1989 | No data for 0-19 yrs |
| 52 | Fricke, 1993 | No data for 0-19 yrs |
| 53 | Fricke, 2002 | No data for 0-19 yrs |
| 54 | Fricke, 2004 | No data for 0-19 yrs |
| 55 | Fricke, 2008 | No data for 0-19 yrs |
| 56 | Gammaitoni, 2003 | No data for 0-19 yrs |
| 57 | Garcia Garcia, 1997 | No data for 0-19 yrs |
| 58 | Garibaldi, 2002 | Use of narcotic analgesic |
| 59 | Garrioch, 1991 | No data for 0-19 yrs |
| 60 | Garwood, 1983 | No data for 0-19 yrs |
| 61 | Gaston, 1984 | Adult patients |
| 62 | Gaston, 1996 | No data for 0-19 yrs |
| 63 | Gatoulis, 2012 | No data for 0-19 yrs |
| 64 | Gazal, 2007 | No data for 0-19 yrs |
| 65 | Gazal, 2017 | No data for 0-19 yrs |
| 66 | Giglio, 1986 | No data for 0-19 yrs |
| 67 | Giglio, 1990 | No data for 0-19 yrs |
| 68 | Giles, 1981 | No data for 0-19 yrs |
| 69 | Giles, 1986 | No data for 0-19 yrs |
| 70 | Gönül, 2015 | Use of narcotic analgesic |
| 71 | Gönül, 2015 | Use of narcotic analgesic |
| 72 | Jain, 1986 | No data for 0-19 yrs |
| 73 | Kara, 2010 | No data for 0-19 yrs |
| 74 | Kiersh, 1993 | No data for 0-19 yrs |
| 75 | Kiersh, 1994 | No data for 0-19 yrs |
| 76 | Laska, 1986 | No data for 0-19 yrs |
| 77 | Leguen, 1985 | No data for 0-19 yrs |
| 78 | Levin, 1997 | No data for 0-19 yrs |
| 79 | Li, 2012 | No data for 0-19 yrs |
| 80 | Lysell, 1992 | No data for 0-19 yrs |
| 81 | Malmstrom, 2002 | No data for 0-19 yrs |
| 82 | Malmstrom, 2004 | No data for 0-19 yrs |
| 83 | Markowitz, 1985 | No data for 0-19 yrs |
| 84 | Markus, 1980 | No data for 0-19 yrs |
| 85 | McGaw, 1987 | Analgesics given after pain had developed |
| 86 | Medve, 2001 | No data for 0-19 yrs |
| 87 | Mehlisch, 2010 | No data for 0-19 yrs |
| 88 | Mehlisch, 1984 | No data for 0-19 yrs |
| 89 | Mehlisch, 1990 | No data for 0-19 yrs |
| 90 | Mehlisch, 1990 | No data for 0-19 yrs |
| 91 | Mehlisch, 1994 | No data for 0-19 yrs |
| 92 | Mehlisch, 1995 | No data for 0-19 yrs |
| 93 | Mehlisch, 2010 | No data for 0-19 yrs |
| 94 | Mehlish, 1998 | No data for 0-19 yrs |
| 95 | Mehrvarzfar, 2012 | Adult patients |
| 96 | Melisch, 2002 | No data for 0-19 yrs |
| 97 | Merry, 2010 | No data for 0-19 yrs |
| 98 | Michael, 2006 | No data for 0-19 yrs |
| 99 | Møller, 2000 | No data for 0-19 yrs |
| 100 | Møller, 2005 | No data for 0-19 yrs |
| 101 | Moore, 1985 | Analgesics given after pain had developed |
| 102 | Moore, 2011 | No data for 0-19 yrs |
| 103 | Morrison, 1999 | No data for 0-19 yrs |
| 104 | Naoumova, 2012 | No RCT |
| 105 | Negm, 1989 | No data for 0-19 yrs |
| 106 | Nelson 1994 | No data for 0-19 yrs |
| 107 | Nelson, 1985 | No data for 0-19 yrs |
| 108 | Nelson, 1994 | No data for 0-19 yrs |
| 109 | Nørholt, 1995 | No data for 0-19 yrs |
| 110 | Nørholt, 1996 | No data for 0-19 yrs |
| 111 | Nørholt, 1998 | No data for 0-19 yrs |
| 112 | Nørholt, 2011 | No data for 0-19 yrs |
| 113 | Ohnishi, 1983 | Drugs requiering prescription |
| 114 | Olmedo, 2001 | No data for 0-19 yrs |
| 115 | Olson, 2001 | No data for 0-19 yrs |
| 116 | Olstad, 1986 | No data for 0-19 yrs |
| 117 | Orozco-Solis, 2016 | No data for 0-19 yrs |
| 118 | Ostenfeld, 2011 | No data for 0-19 yrs |
| 119 | Ozkal, 1996 | Cannot be retrieved |
| 120 | Parirokh, 2010 | No data for 0-19 yrs |
| 121 | Parirokh, 2014 | No data for 0-19 yrs |
| 122 | Pasqualini, 2012 | No data for 0-19 yrs |
| 123 | Patel, 1991 | Treatment under general anesthesia |
| 124 | Patel, 1993 | No data for 0-19 yrs |
| 125 | Paudel, 2010 | No patient-reported outcome |
| 126 | Pearlman, 1997 | Adult patients |
| 127 | Pektas, 2007 | No data for 0-19 yrs |
| 128 | Pendeville, 1995 | Treatment under general anesthesia |
| 129 | Petersen, 1993 | No data for 0-19 yrs |
| 130 | Pierce, 2010 | Analgesics given after pain had developed |
| 131 | Piironen, 1985 | No data for 0-19 yrs |
| 132 | Pouchain, 2015 | No data for 0-19 yrs |
| 133 | Prasanna, 2011 | Adults patients |
| 134 | Qi, 2012 | No data for 0-19 yrs |
| 135 | Quiding, 1982 | No data for 0-19 yrs |
| 136 | Quiding, 2013 | No data for 0-19 yrs |
| 137 | Ragot, 1993 | No data for 0-19 yrs |
| 138 | Ragot, 1994 | No data for 0-19 yrs |
| 139 | Ramanath, 2016 | Adults patients |
| 140 | Reijntjes, 1987 | No data for 0-19 yrs |
| 141 | Reines, 1986 | No data for 0-19 yrs |
| 142 | Roelofse, 1993 | No data for 0-19 yrs |
| 143 | Roelofse, 1996 | No data for 0-19 yrs |
| 144 | Rogers, 1999 | No data for 0-19 yrs |
| 145 | Rømsing, 2004 | No dental treatment |
| 146 | Rondeau, 1980 | Treatment under general anesthesia |
| 147 | Rowe, 1980 | No data for 0-19 yrs |
| 148 | Rowe, 1981 | No data for 0-19 yrs |
| 149 | Schmelzeisen, 1993 | No analgesics administered |
| 150 | Schnitzer, 2005 | No data for 0-19 yrs |
| 151 | Schultze-Mosgau, 1995 | No data for 0-19 yrs |
| 152 | Schwartz, 2007 | No data for 0-19 yrs |
| 153 | Scott, 1986 | No data for 0-19 yrs |
| 154 | Segura, 2000 | No data for 0-19 yrs |
| 155 | Selçuk, 1996 | No data for 0-19 yrs |
| 156 | Selçuk, 1998 | No data for 0-19 yrs |
| 157 | Selimovic. 2011 | No data for 0-19 yrs |
| 158 | Sençift, 1997 | No data for 0-19 yrs |
| 159 | Sener, 2005 | No data for 0-19 yrs |
| 160 | Seveker, 2017 | No analgesics administered |
| 161 | Seymour, 1984 | No data for 0-19 yrs |
| 162 | Seymour, 1986 | Treatment under general anesthesia |
| 163 | Shah, 2010 | No analgesics administered |
| 164 | Sharma, 1994 | No data for 0-19 yrs |
| 165 | Sharpless, 2013 | No analgesics administered |
| 166 | Shimura, 1981 | No data for 0-19 yrs |
| 167 | Siddique, 2015 | Not RCT |
| 168 | Silva de, 2016 | No data for 0-19 yrs |
| 169 | Sindet-Pedersen, 1986 | No data for 0-19 yrs |
| 170 | Sisk, 1989 | Treatment under sedation |
| 171 | Sisk, 1990 | Treatment under sedation |
| 172 | Skjelbred, 1984 | No data for 0-19 yrs |
| 173 | Skoglund, 1984 | No data for 0-19 yrs |
| 174 | Skoglund, 1986 | No data for 0-19 yrs |
| 175 | Skoglund, 1991 | No data for 0-19 yrs |
| 176 | Steffen, 2002 | Only in German |
| 177 | Sunshine, 1983 | No data for 0-19 yrs |
| 178 | Sunshine, 1998 | No data for 0-19 yrs |
| 179 | Swift, 1993 | No data for 0-19 yrs – patients sedated |
| 180 | Symington, 1988 | No data for 0-19 yrs – some patients treated under general anesthesia |
| 181 | Syrjanen, 1981 | No data for 0-19 yrs |
| 182 | Tai, 1992 | Treatment under general anesthesia |
| 183 | Takarada, 1981 | Only in Japanese |
| 184 | Todd, 1985 | No ages specified |
| 185 | Tong, 2012 | Adult patients |
| 186 | Trindade, 2011 | No data for 0-19 yrs |
| 187 | Trindade, 2012 | No data for 0-19 yrs |
| 188 | Troullos, 1990 | No data for 0-19 yrs – patients sedated |
| 189 | Tucci, 2009 | Narrativ review. |
| 190 | Tuffin, 1990 | No data for 0-19 yrs – treatment under general anesthesia |
| 191 | Turcotte, 1986 | Only in French |
| 192 | Tuzuner, 2011 | No data for 0-19 yrs |
| 193 | Utsunomiya, 1983 | Only in Japanese |
| 194 | Van Aken, 2004 | No data for 0-19 yrs |
| 195 | Van Aken, 2004 | No data for 0-19 yrs – treatment under general anesthesia |
| 196 | Varner, 2009 | No data for 0-19 yrs |
| 197 | Voelker, 2016 | No data for 0-19 yrs |
| 198 | Walton, 1993 | No data for 0-19 yrs |
| 199 | Ward, 1988 | No ages specified – not dental treatment |
| 200 | Westhuijazen, 1994 | No data for 0-19 yrs – treatment under general anesthesia |
| 201 | White, 1982 | No data for 0-19 yrs |
| 202 | Williams, 1995 | No data for 0-19 yrs |
| 203 | Winter, 1983 | No data for 0-19 yrs – treatment under general anesthesia |
| 204 | Wright, 2002 | No data for 0-19 yrs – treatment under general anesthesia |
| 205 | Yoshimasu, 1983 | Only in Japanese |
| 206 | Young, 2013 | No data for 0-19 yrs |
| 207 | Yue, 2013 | No data for 0-19 yrs |
| 208 | Yuki, 1980 | Only in Japanese |
| 209 | Zelenakas, 2004 | No data for 0-19 yrs |
| 210 | Ziccardi, 2000 | No data for 0-19 yrs |
| 211 | Zor, 2011 | No data for 0-19 yrs |
| 212 | Zor, 2014 | No data for 0-19 yrs |
| 213 | Zuniga, 2004 | No data for 0-19 yrs |
| 214 | Zuniga, 2010 | No data for 0-19 yrs |
| 215 | Zuniga, 2011 | No data for 0-19 yrs |
| 216 | Zupelari-Goncalves, 2017 | No data for 0-19 yrs |

1. Akbulut N, Üstüner E, Atakan C, Çölok G. Comparison of the effect of naproxen, etodolac and diclofenac on postoperative sequels following third molar surgery: a randomised, double-blind, crossover study. Med Oral Patol Oral Cir Bucal. 2014;19(2):e149-56. doi: 10.4317/medoral.19518. PMID: 24316711
2. Aken H, Thys L, Veekman L, Buerkle H. Assessing analgesia in single and repeated administrations of propacetamol for postoperative pain: comparison with morphine after dental surgery. Anesth Analg. 2004;98(1):159-65. PMID: 14693612
3. [Alpaslan C](https://www-ncbi-nlm-nih-gov.proxy.mau.se/pubmed/?term=Alpaslan%20C%5BAuthor%5D&cauthor=true&cauthor_uid=9569795), [Alpaslan G](https://www-ncbi-nlm-nih-gov.proxy.mau.se/pubmed/?term=Alpaslan%20G%5BAuthor%5D&cauthor=true&cauthor_uid=9569795), [Uğar D](https://www-ncbi-nlm-nih-gov.proxy.mau.se/pubmed/?term=U%C4%9Far%20D%5BAuthor%5D&cauthor=true&cauthor_uid=9569795). Postoperative pain control by single doses of piroxicam administered sublingually and aspirin. J Marmara Univ Dent Fac. 1997;2(4):658-64. PMID: 9569795
4. Arribas AR, Muzyka BC. Pain control in dentistry. Alpha Omegan. 2003;96(4):18-27. PMID: 14983726
5. Arslan H, Topcuoglu HS, Aladag H. Effectiveness of tenoxicam and ibuprofen for pain prevention following endodontic therapy in comparison to placebo: a randomized double-blind clinical trial. J Oral Sci. 2011;53(2):157-61. PMID: 21712619
6. Atkinson HC, Currie J, Moodie J, Carson S, Evans S, Worthington JP, et al. Combination paracetamol and ibuprofen for pain relief after oral surgery: a dose ranging study. Eur J Clin Pharmacol. 2015;71(5):579-87. doi: 10.1007/s00228-015-1827-x. PMID: 25778933
7. Averbuch M, Katzper M. Gender and the placebo analgesic effect in acute pain. Clin Pharmacol Ther. 2001;70(3):287-91. doi: 10.1067/mcp.2001.118366. PMID: 11557917
8. Bailey BM, Zaki G, Rotman H, Woodwards RT. A double-blind comparative study of soluble aspirin and diclofenac dispersible in the control of postextraction pain after removal of impacted third molars. Int J Oral Maxillofac Surg. 1993;22(4):238-41. PMID: 8409568
9. Best AD, Silva RK, Thomson WM, Tong DC, Cameron CM, Silva HL. Efficacy of Codeine When Added to Paracetamol (Acetaminophen) and Ibuprofen for Relief of Postoperative Pain After Surgical Removal of Impacted Third Molars: a Double-Blinded Randomized Control Trial. J Oral Maxillofac Surg. 2017;75(10):2063-9. doi: 10.1016/j.joms.2017.04.045. PMID: 28586638
10. Bjørnsson GA, Haanaes HR, Skoglund LA. Ketoprofen 75 mg qid versus acetaminophen 1000 mg qid for 3 days on swelling, pain, and other postoperative events after third-molar surgery. J Clin Pharmacol. 2003;43(3):305-14. PMID: 12638400
11. Bjørnsson GA, Haanæs HR, Skoglund LA. Naproxen 500 mg bid versus acetaminophen 1000 mg qid: effect on swelling and other acute postoperative events after bilateral third molar surgery. J Clin Pharmacol. 2003;43(8):849-58. PMID: 12953342
12. Björnsson MA, Simonsson US. Modelling of pain intensity and informative dropout in a dental pain model after naproxcinod, naproxen and placebo administration. Br J Clin Pharmacol. 2011;71(6):899-906. doi: 10.1111/j.1365-2125.2011.03924.x. PMID: 21272053
13. Bocanegra M, Seijas A, Gonzalez YM. Effectiveness and tolerability of once-daily nimesulide versus ibuprofen in pain management after surgical extraction of an impacted third molar: a 24-hour, double-blind, randomized, double-dummy, parallel-group study. Curr Ther Res Clin Exp. 2005;66(3):172-80. doi: 10.1016/j.curtheres.2005.06.007. PMID: 24672121
14. Boerlin V, Maeglin B, Hägler W, Kuhn M, Nüesch E. Analgesic activity of propyphenazone in patients with pain following oral surgery. Eur J Clin Pharmacol. 1986;31(2):127-31. PMID: 3492378
15. Bracco P, Debernardi C, Coscia D, Pasqualini D, Pasqualicchio F, Calabrese N. Efficacy of rofecoxib and nimesulide in controlling postextraction pain in oral surgery: a randomised comparative study. Curr Med Res Opin. 2004;20(1):107-12. doi: 10.1185/030079903125002694. PMID: 14741079
16. Brain P, Leyva R, Doyle G, Kellstein D. Onset of analgesia and efficacy of ibuprofen sodium in postsurgical dental pain: a randomized, placebo-controlled study versus standard ibuprofen. Clin J Pain. 2015;31(5):444-50. doi: 10.1097/AJP.0000000000000142. PMID: 25119511
17. Breivik EK, Björnsson GA. Variation in surgical trauma and baseline pain intensity: effects on assay sensitivity of an analgesic trial. Eur J Oral Sci. 1998;106(4):844-52. PMID: 9708687
18. Chang DJ, Bird SR, Bohidar NR, King T. Analgesic efficacy of rofecoxib compared with codeine/acetaminophen using a model of acute dental pain. Oral Surg Oral Med Oral Pathol Oral Radiol Endod. 2005;100(4):e74-80. doi: 10.1016/j.tripleo.2005.04.026. PMID: 16182156
19. Chang DJ, Desjardins PJ, Bird SR, Black P, Chen E, Petruschke RA, et al. Comparison of rofecoxib and a multidose oxycodone/ acetaminophen regimen for the treatment of acute pain following oral surgery: a randomized controlled trial. Curr Med Res Opin. 2004;20(6):939-49. doi: 10.1185/030079904125003863. PMID: 15200753
20. Chang DJ, Desjardins PJ, Chen E, Polis AB, McAvoy M, Mockoviak SH, et al. Comparison of the analgesic efficacy of rofecoxib and enteric-coated diclofenac sodium in the treatment of postoperative dental pain: a randomized, placebo-controlled clinical trial. Clin Ther. 2002;24(4):490-503. PMID: 12017395
21. Chang DJ, Fricke JR, Bird SR, Bohidar NR, Dobbins TW, Geba GP. Rofecoxib versus codeine/acetaminophen in postoperative dental pain: a double-blind,randomized, placebo- and active comparator-controlled clinical trial. Clin Ther. 2001;23(9):1446-55. PMID: 11589259
22. Chopra D, Rehan HS, Mehra P, Kakkar AK. A randomized, double-blind, placebo-controlled study comparing the efficacy and safety of paracetamol, serratiopeptidase, ibuprofen and betamethasone using the dental impaction pain model. Int J Oral Maxillofac Surg. 2009;38(4):350-5. doi: 10.1016/j.ijom.2008.12.013. PMID: 19168326
23. Christensen S, Paluch E, Jayawardena S, Daniels S, Meeves S. Analgesic Efficacy of a New Immediate-Release/Extended-Release Formulation of Ibuprofen: Results From Single- and Multiple-Dose Postsurgical Dental Pain Studies. Clin Pharmacol Drug Dev. 2017;6(3):302-12. doi: 10.1002/cpdd.297. PMID: 27545511
24. Cochrane DJ, Jarvis B, Keating GM. Etoricoxib. Drugs. 2002;62(18):2637-51. doi: 10.2165/00003495-200262180-00006. PMID: 12466002
25. Cooper SA, Engel J, Ladov M, Precheur H, Rosenheck A, Rauch D. Analgesic efficacy of an ibuprofen-codeine combination. Pharmacotherapy. 1982;2(3):162-7. PMID: 6763202
26. Cooper SA, Schachtel BP, Goldman E, Gelb S, Cohn P. Ibuprofen and acetaminophen in the relief of acute pain: a randomized, double-blind, placebo-controlled study. J Clin Pharmacol. 1989;29(11):1026-30. PMID: 2689471
27. Cooper SA. Five studies on ibuprofen for postsurgical dental pain. Am J Med. 1984;77(1A):70-7. PMID: 6465164
28. Coulthard P, Haywood D, Tai MA, Jackson-Leech D, Pleuvry BJ, Macfarlane TV. Treatment of postoperative pain in oral and maxillofacial surgery. Br J Oral Maxillofac Surg. 2000;38(6):588-92. doi: 10.1054/bjom.2000.0536. PMID: 11092771
29. Coulthard P, Hill CM, Frame JW, Barry H, Ridge BD, Bacon TH. Pain control with paracetamol from a sustained release formulation and a standard release formulation after third molar surgery: a randomised controlled trial. Br Dent J. 2001;191(6):319-24. doi: 10.1038/sj.bdj.4801174a. PMID: 11587503
30. Daniels S, Reader S, Berry P, Goulder M. Onset of analgesia with sodium ibuprofen, ibuprofen acid incorporating poloxamer and acetaminophen-a single-dose, double-blind, placebo-controlled study in patients with post-operative dental pain. Eur J Clin Pharmacol. 2009;65(4):343-53. doi: 10.1007/s00228-009-0614-y. PMID: 19252905
31. Daniels SE, Bandy DP, Christensen SE, Boice J, Losada MC, Liu H, et al. Evaluation of the dose range of etoricoxib in an acute pain setting using the postoperative dental pain model. Clin J Pain. 2011;27(1):1-8. PMID: 21188849
32. Daniels SE, Desjardins PJ, Bird SR, Smugar SS, Tershakovec AM. Rofecoxib 50 mg and valdecoxib 20 or 40 mg in adults and adolescents with postoperative pain after third molar extraction: results of two randomized, double-blind, placebocontrolled, single-dose studies. Clin Ther. 2006;28(7):1022-34. doi: 10.1016/j.clinthera.2006.07.005. PMID: 16990080
33. Daniels SE, Goulder MA, Aspley S, Reader S. A randomised, five-parallelgroup, placebo-controlled trial comparing the efficacy and tolerability of analgesic combinations including a novel single-tablet combination of ibuprofen/paracetamol for postoperative dental pain. Pain. 2011;152(3):632-42. doi: 10.1016/j.pain.2010.12.012. PMID: 21257263
34. Desjardins PJ, Cooper SA, Gallegos TL, Allwein JB, Reynolds DC, Kruger GO, et al. The relative analgesic efficacy of propiram fumarate, codeine, aspirin, and placebo in post-impaction dental pain. J Clin Pharmacol. 1984;24(1):35-42. PMID: 6368614
35. Desjardins PJ, Cooper SA, Ruderman CM, Gallegos LT, Reynolds DC, Kruger GO. The effects of fendosal, aspirin and placebo on postoperative dental pain. A dose-ranging and efficacy study. Pharmacotherapy 1983;3(1):52-7. PMID: 6344034
36. Dietrich EM, Griessinger N, Neukam FW, Schlittenbauer T. Consultation with a specialized pain clinic reduces pain after oral and maxillofacial surgery. J Cranio-Maxillofac Surg. 2017;45(2):281-9. doi: 10.1016/j.jcms.2016.12.009. PMID: 28057402
37. Dionne RA, Wirdzek PR, Fox PC, Dubner R. Suppression of postoperative pain by the combination of a nonsteroidal anti-inflammatory drug, flurbiprofen, and a longacting local anesthetic, etidocaine. J Am Dent Assoc. 1984;108(4):598-601. PMID: 6586802
38. Dodd MD, Graham CA. Unintentional overdose of analgesia secondary to acute dental pain. Br Dent J. 2002;193(4):211-2. PMID: 12222908
39. Doyle G, Jayawardena S, Ashraf E, Cooper SA. Efficacy and tolerability of nonprescription ibuprofen versus celecoxib for dental pain. J Clin Pharmacol. 2002;42(8):912-9. PMID: 12162474
40. Edmondson HD, Bradshaw AJ. Analgesia following oral surgery: a comparative study of Solpadeine and a soluble form of dextropropoxyphene napsylate and paracetamol. J Int Med Res. 1983;11(4):228-31. doi: 10.1177/030006058301100406. PMID: 6137427
41. El Batawi HY. Effect of intraoperative analgesia on children's pain perception during recovery after painful dental procedures performed under general anaesthesia. Eur Arch Paediatr Dent. 2015;16(1):35-41. doi: 10.1007/s40368-014-0143-y. PMID: 25260983
42. Elhakim M. Painless dental extraction in children. Anaesthesiol Reanim. 1993;18(3):80-2. PMID: 8216665
43. Eroglu CN, Durmus E, Kiresi D. Effect of low-dose dexketoprofen trometamol and paracetamol on postoperative complications after impacted third molar surgery on healthy volunteers: A pilot study. Med Oral Patol Oral Cir Bucal. 2014;19(6):e622-e7. doi: 10.4317/medoral.19835. PMID: 25129247
44. Esteller-Martínez V, Paredes-García J, Valmaseda-Castellón E, Berini-Aytés L, Gay-Escoda C. Analgesic efficacy of diclofenac sodium versus ibuprofen following surgical extraction of impacted lower third molars. Med Oral Patol Oral Cir Bucal. 2004;9(5):448-53; 444-8. PMID: 15580123
45. Forbes JA, Barkaszi BA, Ragland RN, Hankle JJ. Analgesic effect of fendosal, ibuprofen and aspirin in postoperative oral surgery pain. Pharmacotherapy. 1984;4(6):385-91. PMID: 6393076
46. Forbes JA, Beaver WT, Jones KF, Edquist IA, Gongloff CM, Smith WK, et al. Analgesic efficacy of bromfenac, ibuprofen, and aspirin in postoperative oral surgery pain. Clin Pharmacol Ther. 1992;51(3):343-52. doi: 10.1038/clpt.1992.31. PMID: 1544291
47. Forbes JA, Edquist IA, Smith FG, Schwartz MK, Beaver WT. Evaluation of bromfenac, aspirin, and ibuprofen in postoperative oral surgery pain. Pharmacotherapy. 1991;11(1):64-70. PMID: 2020613
48. Forbes JA, Keller CK, Smith JW, Zeleznock JR, Sevelius H, Beaver WT. Analgesic effect of naproxen sodium, codeine, a naproxen-codeine combination and aspirin on the postoperative pain of oral surgery. Pharmacotherapy. 1986;6(5):211-8. PMID: 3540871
49. Forbes JA, White RW, White EH, Hughes MK. An Evaluation of the Analgesic Efficacy of Proquazone and Aspirin in Postoperative Dental Pain. J Clin Pharmacol. 1980;20(7):465-74. PMID: 7000855
50. Forbes JA, Yorio CC, Selinger LR, Rosenmertz SK, Beaver WT. An evaluation of flurbiprofen, aspirin, and placebo in postoperative oral surgery pain. Pharmacotherapy. 1989;9(2):66-73. PMID: 2657675
51. Frame JW, Evans CR, Flaum GR, Langford R, Rout PG. A comparison of ibuprofen and dihydrocodeine in relieving pain following wisdom teeth removal. Br Dent J. 1989;166(4):121-4. PMID: 2920133
52. Frame JW, Rout PGJ. A comparison of the analgesic efficacy of flurbiprofen, diclofenac, dihydrocodeine/paracetamol and placebo following oral surgery. Br J Clin Pract. 1986;40(11):463-7. PMID: 3307859
53. Fricke J, Davis N, Yu V, Krammer G. Lumiracoxib 400 mg compared with celecoxib 400 mg and placebo for treating pain following dental surgery: a randomized, controlled trial. J Pain. 2008;9(1):20-7. doi: 10.1016/j.jpain.2007.08.004. PMID: 17933588
54. Fricke JR, Halladay SC, Francisco CA. Efficacy and safety of naproxen sodium and ibuprofen for pain relief after oral surgery. Current therapeutic research - clinical and experimental. 1993;54(6):619-27. doi: 10.1016/s0011-393x(05)80692-7
55. Fricke JR, Hewitt DJ, Jordan DM, Fisher A, Rosenthal NR. A double-blind placebo-controlled comparison of tramadol/acetaminophen and tramadol in patients with postoperative dental pain. Pain. 2004;109(3):250-7. doi: 10.1016/j.pain.2004.01.004. PMID: 15157685
56. Fricke JR, Karim R, Jordan D, Rosenthal N. A double-blind, single-dose comparison of the analgesic efficacy of tramadol/acetaminophen combination tablets, hydrocodone/acetaminophen combination tablets, and placebo after oral surgery. Clin Ther 2002;24(6):953-68. PMID: 12117085
57. Gammaitoni AR, Galer BS, Bulloch S, Lacouture P, Caruso F, Ma T, et al. Randomized, double-blind, placebo-controlled comparison of the analgesic efficacy of oxycodone 10 mg/acetaminophen 325 mg versus controlled-release oxycodone 20 mg in postsurgical pain. J Clin Pharmacol. 2003;43(3):296-304. PMID: 12638399
58. Garcia Garcia A, Gude Sampedro F, Gandara Rey J, Gallas Torreira M. Trismus and pain after removal of impacted lower third molars. J Oral Maxillofac Surg. 1997;55(11):1223-6. PMID: 9371111
59. Garibaldi JA, Elder MF. Evaluation of ketorolac (Toradol) with varying amounts of codeine for postoperative extraction pain control. Int J Oral Maxillofac Surg. 2002;31(3):276-80. PMID: 12190134
60. Garrioch MA, Wardall GJ, Fitch W. A comparison of azapropazone and aspirin for pain relief following dental extractions. Anaesthesia. 1991;46(10):828-32. PMID: 1842215
61. Garwood AJ, Lownie JF, Cleaton-Jones PE, Butz SJ. The effect of Ibuprofen (Brufen) following the removal of impacted third molar teeth. J Dent Assoc S Afr. 1983;38(12):739-42. PMID: 6588641
62. Gaston G. A double-blind, randomized, parallel-group study of the pharmacokinetics and onset of action of Naprelan in patients following oral surgery. Am J Orthop. 1996;25(9 Suppl):37-41. PMID: 8886216
63. Gaston GW, Mallow RD, Frank JE. The efficacy of etodolac for patients with pain following oral surgery. J Oral Maxillofac Surg. 1984;42(6):362-6. PMID: 6232359
64. Gatoulis SC, Voelker M, Fisher M. Assessment of the efficacy and safety profiles of aspirin and acetaminophen with codeine: results from 2 randomized, controlled 560 trials in individuals with tension-type headache and postoperative dental pain. Clin Ther 2012;34(1):138-48. doi: 10.1016/j.clinthera.2011.11.018. PMID: 22169050
65. Gazal G, Al-Samadani KH. Comparison of paracetamol, ibuprofen, and diclofenac potassium for pain relief following dental extractions and deep cavity preparations. Saudi Med J. 2017;38(3):284-91. doi: 10.15537/smj.2017.3.16023. PMID: 28251224
66. Gazal G, Mackie IC. A comparison of paracetamol, ibuprofen or their combination for pain relief following extractions in children under general anaesthesia: a randomized controlled trial. International journal of paediatric dentistry. 2007;17(3):169-77. doi: 10.1111/j.1365-263X.2006.00806.x. PMID: 17397460
67. Giglio JA, Campbell RL. Comparison of etodolac, zomepirac, and placebo for relief of pain after oral surgery. J Oral Maxillofac Surg. 1986;44(10):765-70. PMID: 2944997
68. Giglio JA, Laskin DM. Double-blind comparison of meclofenamate sodium plus codeine, meclofenamate sodium, codeine, and placebo for relief of pain following surgical removal of third molars. J Oral Maxillofac Surg. 1990;48(8):785-90. PMID: 2197381
69. Giles AD, Hill CM, Shepherd JP, Stewart DJ, Pickvance NJ. A single dose assessment of an ibuprofen/codeine combination in postoperative dental pain. Int J Oral Maxillofac Surg. 1986;15(6):727-32. PMID: 3100675
70. Giles AD. Analgesia following dental surgery: a comparison of brufen and distalgesic. Br J Oral Surg. 1981;19(2):105-11. PMID: 6942875
71. Gönül O, Satilmış T, Bayram F, Göçmen G, Sipahi A, Göker K.. Effect of submucosal application of tramadol on postoperative pain after third molar surgery. Head Face Med. 2015;11:35. doi: 10.1186/s13005-015-0090-9. PMID: 26467984
72. Gönül O, Satilmiş T, Ciftci A, Sipahi A, Garip H, Göker K. Comparison of the Effects of Topical Ketamine and Tramadol on Postoperative Pain After Mandibular Molar Extraction. J Oral Maxillofac Surg. 2015;73(11):2103-7. doi: 10.1016/j.joms.2015.05.012. PMID: 26044602
73. Jain AK, Ryan JR, McMahon FG, Kuebel JO, Walters PJ, Noveck C. Analgesic efficacy of low-dose ibuprofen in dental extraction pain. Pharmacotherapy. 1986;6(6):318-22. PMID: 3547351
74. Kara IM, Polat S, Inci MF, Gümüş C. Analgesic and anti-inflammatory effects of oxaprozin and naproxen sodium after removal of impacted lower third molars: a randomized, double-blind, placebo-controlled crossover study. J Oral Maxillofac Surg. 2010;68(5):1018-24. doi: 10.1016/j.joms.2009.09.094. PMID: 20206429
75. Kiersch TA, Halladay SC, Hormel PC. A single-dose, double-blind comparison of naproxen sodium, acetaminophen, and placebo in postoperative dental pain. Clin Ther. 1994;16(3):394-404. PMID: 7923306
76. Kiersch TA, Halladay SC, Koschik M. A double-blind, randomized study of naproxen sodium, ibuprofen, and placebo in postoperative dental pain. Clin Ther. 1993;15(5):845-54. PMID: 8269451
77. Laska EM, Sunshine A, Marrero I, Olson N, Siegel C, McCormick N. The correlation between blood levels of ibuprofen and clinical analgesic response. Clin Pharmacol Ther. 1986;40(1):1-7. doi: 10.1038/clpt.1986.129. PMID: 3522030
78. Leguen MA. Single-blind clinical trial comparing use of fentiazac and paracetamol in postendodontic periodontitis. Clin Ther. 1985;7(2):145-50. PMID: 3886139
79. Levin LM, Cooper SA, Betts NJ, Wedell D, Hermann DG, Lamp C, et al. Ketoprofen Dental Pain Study. J Clin Dent. 1997;8(4):103-6. PMID: 26630719
80. Li H, Mandema J, Wada R, Jayawardena S, Desjardins P, Doyle G, et al. Modeling the onset and offset of dental pain relief by ibuprofen. J Clin Pharmacol. 2012;52(1):89-101. doi: 10.1177/0091270010389470. PMID: 21383341
81. Lysell L, Anzén B. Pain control after third molar surgery - a comparative study of ibuprofen (Ibumetin) and a paracetamol/codeine combination (Citodon). Swed Dent J. 1992;16(4):151-60. PMID: 1455326
82. Malmstrom K, Fricke JR, Kotey P, Kress B, Morrison B. A comparison of rofecoxib versus celecoxib in treating pain after dental surgery: a single-center, randomized, double-blind, placebo- and active-comparator-controlled, parallel-group, single-dose study using the dental impaction pain model. Clin Ther. 2002;24(10):1549-60. PMID: 12462285
83. Malmstrom K, Sapre A, Couglin H, Agrawal NG, Mazenko RS, Fricke JR. Etoricoxib in acute pain associated with dental surgery: a randomized, double-blind, placebo- and active comparator-controlled dose-ranging study. Clin Ther. 2004;26(5):667-79. PMID: 15220011
84. Markowitz NR, Young SK, Rohrer MD, Turner JL. Comparison of meclofenamate sodium with buffered aspirin and placebo in the treatment of postsurgical dental pain. J Oral Maxillofac Surg. 1985;43(7):517-22. PMID: 3859595
85. Markus AF, Gough D. A clinical trial of Suprofen and aspirin in postoperative dental pain. Int J Oral Surg. 1980;9(6):477-9. PMID: 6783576
86. McGaw T, Raborn W, Grace M. Analgesics in pediatric dental surgery: relative efficacy of aluminum ibuprofen suspension and acetaminophen elixir. ASDC J Dent Child. 1987;54(2):106-9. PMID: 3470325
87. Medve RA, Wang J, Karim R. Tramadol and acetaminophen tablets for dental pain. Anesth Prog. 2001;48(3):79-81. PMID: 11724223
88. Mehlisch DR, Ardia A, Pallotta T. A controlled comparative study of ibuprofen arginate versus conventional ibuprofen in the treatment of postoperative dental pain. J Clin Pharmacol. 2002;42(8):904-11. PMID: 12162473
89. Mehlisch DR, Aspley S, Daniels SE, Bandy DP. Comparison of the analgesic efficacy of concurrent ibuprofen and paracetamol with ibuprofen or paracetamol alone in the management of moderate to severe acute postoperative dental pain in adolescents and adults: a randomized, double-blind, placebo-controlled, parallel-group, single-dose, two-center, modified factorial study. Clin Ther. 2010;32(5):882-95. doi: 10.1016/j.clinthera.2010.04.022. PMID: 20685496
90. Mehlisch DR, Aspley S, Daniels SE, Southerden KA, Christensen KS. A single-tablet fixed-dose combination of racemic ibuprofen/paracetamol in the management of moderate to severe postoperative dental pain in adult and adolescent patients: a multicenter, two-stage, randomized, double-blind, parallel-group, placebo-controlled, factorial study. Clin Ther. 2010;32(6):1033-49. doi: 10.1016/j.clinthera.2010.06.002. PMID: 20637958
91. Mehlisch DR, Brown P. Single-dose therapy with diclofenac potassium, aspirin, or placebo following dental impaction surgery. Today's therapeutic trends. 1994;12:15-31.
92. Mehlisch DR, Frakes LA. A controlled comparative evaluation of acetaminophen and aspirin in the treatment of postoperative pain. Clin Ther. 1984;7(1):89-97. PMID: 6394131
93. Mehlisch DR, Jasper RD, Brown P, Korn SH, McCarroll K, Murakami AA. Comparative study of ibuprofen lysine and acetaminophen in patients with postoperative dental pain. Clin Ther. 1995;17(5):852-60. PMID: 8595637
94. Mehlisch DR, Sollecito WA, Helfrick JF, Leibold DG, Markowitz R, Schow CE, et al. Multicenter clinical trial of ibuprofen and acetaminophen in the treatment of postoperative dental pain. J Am Dent Assoc. 1990;121(2):257-63. PMID: 2205641
95. Mehlisch DR, Sterling WR, Mazza FA, Singer JM. A single-dose study of the efficacy and safety of FS 205-397 (250 mg or 500 mg) versus aspirin and placebo in the treatment of postsurgery dental pain. J Clin Pharmacol. 1990;30(9):815-23. PMID: 2277129
96. Mehlisch DR. Double-blind, single-dose comparison of bromfenac sodium, tramadol, and placebo after oral surgery. J Clin Pharmacol. 1998;38(5):455-62. PMID: 9602960
97. Mehrvarzfar P, Abbott PV, Saghiri MA, Delvarani A, Asgar K, Lotfi M, et al. Effects of three oral analgesics on postoperative pain following root canal preparation: a controlled clinical trial. Int Endod J. 2012;45(1):76-82. doi: 10.1111/j.1365-2591.2011.01950.x . PMID: 21902704
98. Merry AF, Gibbs RD, Edwards J, Ting GS, Frampton C, Davies E, et al. Combined acetaminophen and ibuprofen for pain relief after oral surgery in adults: a randomized controlled trial. Br J Anaesth. 2010;104(1):80-8. doi: 10.1093/bja/aep338. PMID: 20007794
99. Michael HC, Sindet-Pederson S, Seymour RA, Hawkesford JE, Coulthard P, Lamey PJ, et al. Analgesic efficacy of the cyclooxygenase-inhibiting nitric oxide donor AZD3582 in postoperative dental pain: comparison with naproxen and rofecoxib in two randomized, double-blind, placebo-controlled studies. Clin Ther. 2006;28(9):1279-95. doi: 10.1016/j.clinthera.2006.09.015. PMID: 17062301
100. Møller PL, Juhl GI, Payen-Champenois C, Skoglund LA. Intravenous acetaminophen (paracetamol): comparable analgesic efficacy, but better local safety than its prodrug, propacetamol, for postoperative pain after third molar surgery. Anesth Analg. 2005;101(1):90-6, table of contents. doi: 10.1213/01.ANE.0000155297.47955.D6. PMID: 15976212
101. Møller PL, Nørholt SE, Ganry HE, Insuasty JH, Vincent FG, Skoglund LA, et al. Time to onset of analgesia and analgesic efficacy of effervescent acetaminophen 1000 mg compared to tablet acetaminophen 1000 mg in postoperative dental pain: a single-dose, double-blind, randomized, placebo-controlled study. J Clin Pharmacol. 2000;40(4):370-8. PMID: 10761164
102. Moore PA, Acs G, Hargreaves JA. Postextraction pain relief in children: a clinical trial of liquid analgesics. Int J Clin Pharmacol Ther Toxicol. 1985;23(11):573-7. PMID: 3908330
103. Moore RA, Straube S, Paine J, Derry S, McQuay HJ. Minimum efficacy criteria for comparisons between treatments using individual patient meta-analysis of acute pain trials: Examples of etoricoxib, paracetamol, ibuprofen, and ibuprofen/paracetamol combinations after third molar extraction. Pain. 2011;152(5):982-9. doi: 10.1016/j.pain.2010.11.030. PMID: 21414722
104. Morrison BW, Christensen S, Yuan W, Brown J, Amlani S, Seidenberg B. Analgesic efficacy of the cyclooxygenase-2-specific inhibitor rofecoxib in post-dental surgery pain: a randomized, controlled trial. Clin Ther. 1999;21(6):943-53. doi: 10.1016/S0149-2918(99)80016-2. PMID: 10440619
105. Naoumova J, Kjellberg H, Kurol J, Mohlin B. Pain, discomfort, and use of analgesics following the extraction of primary canines in children with palatally displaced canines. Int J Paediatr Dent. 2012;22(1):17-26. doi: 10.1111/j.1365-263X.2011.01152.x. PMID: 21689179
106. Negm MM. Management of endodontic pain with nonsteroidal anti-inflammatory agents: a double-blind, placebo-controlled study. Oral Surg Oral Med Oral Pathol. 1989;67(1):88-95. PMID: 2911450
107. Nelson S, Brahim J. An evaluation of the analgesic efficacy of diclofenac potassium, aspirin, and placebo in postoperative dental pain. Today's therapeutic trends. 1994;12:3-14.
108. Nelson SL, Bergman SA. Relief of dental surgery pain: a controlled 12-hour comparison of etodolac, aspirin, and placebo. Anesth Prog. 1985;32(4):151-6. PMID: 2934008
109. Nelson SL, Brahim JS, Korn SH, Greene SS, Suchower LJ. Comparison of single-dose ibuprofen lysine, acetylsalicylic acid, and placebo for moderate-to-severe postoperative dental pain. Clin Ther. 1994;16(3):458-65. PMID: 7923312
110. Nørholt SE, Aagaard E, Svensson P, Sindet-Pedersen S. Evaluation of trismus, bite force, and pressure algometry after third molar surgery: a placebo-controlled study of ibuprofen. J Oral Maxillofac Surg. 1998;56(4):420-7; discussion 427-9. PMID: 9541340
111. Nørholt SE, Hallmer F, Hartlev J, Pallesen L, Blomlöf J, Hansen EJ, et al. Analgesic efficacy with rapidly absorbed ibuprofen sodium dihydrate in postsurgical dental pain: results from the randomized QUIKK trial. Int J Clin Pharmacol Ther. 2011;49(12):722-9. doi: 10.5414/cp201553. PMID: 22122814
112. Nørholt SE, Sindet-Pedersen S, Bugge C, Branebjerg PE, Ersbøll BK, Bastian HL. A randomized, double-blind, placebo-controlled, dose-response study of the analgesic effect of lornoxicam after surgical removal of mandibular third molars. J Clin Pharmacol. 1995;35(6):606-14. PMID: 7665721
113. Nørholt SE, Sindet-Pedersen S, Larsen U, Bang U, Ingerslev J, Nielsen O, et al. Pain control after dental surgery: a double-blind, randomised trial of lornoxicam versus morphine. Pain. 1996;67(2-3):335-43. PMID: 8951927
114. Ohnishi M, Kawai T, Ogawa N. Double-blind evaluation of piroxicam and indomethacin in the treatment of inflammation following oral surgery. Eur J Rheumatol Inflamm. 1983;6(3):259-65. PMID: 6391930
115. Olmedo MV, Gálvez R, Vallecillo M. Double-blind parallel comparison of multiple doses of ketorolac, ketoprofen and placebo administered orally to patients with postoperative dental pain. Pain. 2001;90(1-2):135-41. PMID: 11166979
116. Olson NZ, Otero AM, Marrero I, Tirado S, Cooper S, Doyle G, et al. Onset of analgesia for liquigel ibuprofen 400 mg, acetaminophen 1000 mg, ketoprofen 25 mg, and placebo in the treatment of postoperative dental pain. J Clin Pharmacol. 2001;41(11):1238-47. PMID: 11697757
117. Olstad OA, Skjelbred P. The effects of indoprofen vs paracetamol on swelling, pain and other events after surgery. Int J Clin Pharmacol Ther Toxicol. 1986;24(1):34-8. PMID: 3514486
118. Orozco-Solís M, García-Ávalos Y, Pichardo-Ramírez C, Tobías-Azúa F, Zapata-Morales JR, Aragon-Martínez OH, et al. Single dose of diclofenac or meloxicam for control of pain, facial swelling, and trismus in oral surgery. Med Oral Patol Oral Cir Bucal. 2016;21(1):e127-34. doi: 10.4317/medoral.20925. PMID: 26615509
119. Ostenfeld T, Price J, Albanese M, Bullman J, Guillard F, Meyer I, et al. A randomized, controlled study to investigate the analgesic efficacy of single doses of the cannabinoid receptor-2 agonist GW842166, ibuprofen or placebo in patients with acute pain following third molar tooth extraction. Clin J Pain. 2011;27(8):668-76. doi: 10.1097/AJP.0b013e318219799a. PMID: 21540741
120. Ozkal S, Gurbuzer B, Dogan N, Kizilkaya E, Yucel O. Clinical and ultrasonographic evaluation of the effect of two non-opioid analgesic for postoperative pain and edema. Agri dergisi. 1996;8:31-7.
121. Parirokh M, Ashouri R, Rekabi AR, Nakhaee N, Pardakhti A, Askarifard S, et al. The effect of premedication with ibuprofen and indomethacin on the success of inferior alveolar nerve block for teeth with irreversible pulpitis. J Endod. 2010;36(9):1450-4. doi: 10.1016/j.joen.2010.05.007. PMID: 20728707
122. Parirokh M, Sadr S, Nakhaee N, Abbott PV, Manochehrifar H. Comparison between prescription of regular or on-demand ibuprofen on postoperative pain after singlevisit root canal treatment of teeth with irreversible pulpitis. J Endod. 2014;40(2):151-4. doi: 10.1016/j.joen.2013.09.024. PMID: 24461395
123. Pasqualini D, Mollo L, Scotti N, Cantatore G, Castellucci A, Migliaretti G, et al. Postoperative pain after manual and mechanical glide path: a randomized clinical trial. J Endod. 2012;38(1):32-6. doi: 10.1016/j.joen.2011.09.017. PMID: 22152616
124. Patel A, Skelly AM, Kohn H, Preiskel HW. Double-blind placebo-controlled comparison of the analgesic effects of single doses of lornoxicam and aspirin in patients with postoperative dental pain. Br Dent J. 1991;170(8):295-9. PMID: 2036277
125. Patel R. Management of painful inflammatory disorders in paediatric practice. Indian Pract. 1993;46:513-20.
126. Paudel KR, Sah NK, Jaiswal AK. Prevalence of pharmacotherapy in the department of paediatric dentistry. Kathmandu Univ Med J. 2010;8(30):190-4. PMID: 21209533
127. Pearlman B, Boyatzis S, Daly C, Evans R, Gouvoussis J, Highfield J, et al. The analgesic efficacy of ibuprofen in periodontal surgery: a multicentre study. Aust Dent J. 1997;42(5):328-34. PMID: 9409050
128. Pektas ZO, Sener M, Bayram B, Eroglu T, Bozdogan N, Donmez A, et al. A comparison of pre-emptive analgesic efficacy of diflunisal and lornoxicam for postoperative pain management: a prospective, randomized, single-blind, crossover study. Int J Oral Maxillofac Surg. 2007;36(2):123-7. doi: 10.1016/j.ijom.2006.10.005. PMID: 17157478
129. Pendeville PE, Boven MJ, Contreras V, Scholtes JL, Fosseur G, Lechien P, et al. Ketorolac tromethamine for postoperative analgesia in oral surgery. Acta Anaesthesiol Belg. 1995;46(1):25-30. PMID: 7618425
130. Petersen JK, Hansson F, Strid S. The effect of an ibuprofen-codeine combination for the treatment of patients with pain after removal of lower third molars. J Oral Maxillofac Surg. 1993;51(6):637-40. PMID: 8492200
131. Pierce CA, Voss B. Efficacy and safety of ibuprofen and acetaminophen in children and adults: A meta-analysis and qualitative review. Ann Pharmacother. 2010;44(3):489-506. doi: 10.1345/aph.1M332. PMID: 20150507
132. Piironen J, Sjoblad AM, Oikarinen VJ. The analgesic efficacy of one pre- and postoperative dose of piroxicam in oral surgery. Proc Finn Dent Soc. 1985;81(5-6):271-4. PMID: 3878518
133. Pouchain EC, Costa FWG, Bezerra TP, Soares ECS. Comparative efficacy of nimesulide and ketoprofen on inflammatory events in third molar surgery: a split-mouth, prospective, randomized, double-blind study. Int J Oral Maxillofac Surg. 2015;44(7):876-84. doi: 10.1016/j.ijom.2014.10.026. PMID: 25847016
134. Prasanna N, Subbarao CV, Gutmann JL. The efficacy of pre-operative oral medication of lornoxicam and diclofenac potassium on the success of inferior alveolar nerve block in patients with irreversible pulpitis: a double-blind, randomised controlled clinical trial. Int Endod J. 2011;44(4):330-6. PMID: 21692235
135. Qi DS, May LG, Zimmerman B, Peng P, Atillasoy E, Brown JD, et al. A randomized, double-blind, placebo-controlled study of acetaminophen 1000 mg versus acetaminophen 650 mg for the treatment of postsurgical dental pain. Clin Ther. 2012;34(12):2247-58.e3. doi: 10.1016/j.clinthera.2012.11.003. PMID: 23200183
136. Quiding H, Jonzon B, Svensson O, Webster L, Reimfelt A, Karin A, et al. TRPV1 antagonistic analgesic effect: a randomized study of AZD1386 in pain after third molar extraction. Pain. 2013;154(6):808-12. doi: 10.1016/j.pain.2013.02.004. PMID: 23541425
137. Quiding H, Oikarinen V, Huitfeldt B, Koskimo M, Leikomaa H, Nyman C. An analgesic study with repeated doses of phenazone, phenazone plus dextropropoxyphene, and paracetamol, using a visual analogue scale. Int J Oral Surg. 1982;11(5):304-9. PMID: 6818169
138. Ragot JP, Giorgi M, Marinoni M, Macchi M, Mazza P, Rizzo S, et al. Acute activity of nimesulide in the treatment of pain after oral surgery - Double blind, placebo and mefenamic acid controlled study. Eur J Clin Res. 1994;5:39-50.
139. Ragot JP, Monti T, Macciocchi A. Controlled clinical investigation of acute analgesic activity of nimesulide in pain after oral surgery. Drugs. 1993;46(Suppl 1):162-7. doi: 10.2165/00003495-199300461-00039. PMID: 7506161
140. Ramanath KV, Singh R, Shaj AV, Gopinath D, Chacko SM, Chama S. Study on drug utilization pattern in Dental Department of a Tertiary Care Teaching Hospital: A prospective study. Res J Pharm Biol Chem Sci. 2016;7:319-27.
141. Reijntjes RJ, Boering G, Wesseling H, Rijn LJ. Suprofen versus paracetamol after oral surgery. Int J Oral Maxillofac Surg. 1987;16(1):45-9. PMID: 3104494
142. Reines HD, Hunt P, Rambo W, Loadholt CB. Oral ciramadol: a new analgesic for postoperative pain. J Clin Pharmacol. 1986;26(2):111-4. PMID: 3753988
143. Roelofse JA, Bijl P, Joubert JJ. An open comparative study of the analgesic effects of tenoxicam and diclofenac sodium after third molar surgery. Anesth Pain Control Dent. 1993;2(4):217-22. PMID: 8180524
144. Roelofse JA, Bijl P, Joubert JJ. Analgesic and anti-inflammatory efficacy of tenoxicam and diclofenac sodium after third molar surgery. Anesth Prog. 1996;43(4):103-7. PMID: 10323115
145. Rogers MJ, Johnson BR, Remeikis NA, BeGole EA. Comparison of effect of intracanal use of ketorolac tromethamine and dexamethasone with oral ibuprofen on post treatment endodontic pain. J Endod. 1999;25(5):381-4. doi: 10.1016/S0099-2399(06)81176-3. PMID: 10530266
146. Rømsing J, Møiniche S. A systematic review of COX-2 inhibitors compared with traditional NSAIDs, or different COX-2 inhibitors for post-operative pain. Acta Anaesthesiol Scand. 2004;48(5):525-46. doi: 10.1111/j.0001-5172.2004.00379.x. PMID: 15101847
147. Rondeau PL, Yeung E, Nelson P. Dental surgery pain analgesic. J Can Dent Assoc. 1980;46(7):433-9. PMID: 6992954
148. Rowe NH, Cudmore CL, Turner JL. Control of pain by mefenamic acid following removal of impacted molar. A double-blind, placebo-controlled study. Oral Surg Oral Med Oral Pathol. 1981;51(6):575-80. PMID: 7019803
149. Rowe NH, Shekter MA, Turner JL, Spencer J, Dowson J, Petrick TJ. Control of pain resulting from endodontic therapy: a double-blind, placebo-controlled study. Oral Surg Oral Med Oral Pathol. 1980;50(3):257-63. PMID: 6997792
150. Schmelzeisen R, Frolich JC. Prevention of postoperative swelling and pain by dexamethasone after operative removal of impacted third molar teeth. Eur J Clin Pharmacol. 1993;44(3):275-7. PMID: 8491244
151. Schnitzer TJ, Gitton X, Jayawardene S, Sloan VS. Lumiracoxib in the treatment of osteoarthritis, rheumatoid arthritis and acute postoperative dental pain: results of three dose-response studies. Curr Med Res Opin. 2005;21(1):151-61. PMID: 15881487
152. Schultze-Mosgau S, Schmelzeisen R, Frölich JC, Schmele H. Use of ibuprofen and methylprednisolone for the prevention of pain and swelling after removal of impacted third molars. Journal of oral and maxillofacial surgery. 1995;53(1):2-7; discussion 7-8. PMID: 7799116
153. Schwartz JI, Kotey PN, Fricke JR, Gottesdiener K. MK-0703 (a cyclooxygenase-2 inhibitor) in acute pain associated with dental surgery: a randomized, double-blind, placebo- and active comparator-controlled dose-ranging study. Am J Ther. 2007;14(1):13-9. doi: 10.1097/MJT.0b013e31802dfb62. PMID: 17303970
154. Scott R, Ellis E, Upton LG. Double-blind evaluation of etodolac (200 mg, 400 mg) compared with zomepirac (100 mg) and placebo on third molar extraction pain. Oral Surg Oral Med Oral Pathol. 1986;62(6):638-42. PMID: 2948143
155. Segura JJ, Baldizon-Rodriguez C, Toledo-Balladares S, Flores-Treviño JJ, Calzado-Flores C. A new therapeutic scheme of ibuprofen to treat postoperatory endodontic dental pain. Proc West Pharmacol Soc. 2000;43:89-91. PMID: 11056966
156. Selcuk E, Gomel M, Apaydin S, Köse T, Tuglular I. The postoperative analgesic efficacy and safety of piroxicam (FDDF) and naproxen sodium. Int J Clin Pharmacol Res. 1998;18(3):21-9. PMID: 9604731
157. Selçuk E, Gomel M, Bellibas SE, Köse T, Tuglular I. Comparison of the analgesic effects of diflunisal and paracetamol in the treatment of postoperative dental pain. Int J Clin Pharmacol Res. 1996;16(2-3):57-65. PMID: 9063757
158. Selimovic E, Ibrahimagic-Seper L, Petricevic N, Nola-Fuchs P. Pain relieve after impacted wisdom teeth extraction dependent on the drug therapy. Coll Antropol. 2011;35(1):133-6. PMID: 21667538
159. Sençift K, Kir S, Tuncer M. Clinical effects of diflunisal and paracetamol in impacted mandibular third molar surgery. J Marmara Univ Dent Fac. 1997;2(4):673-81. PMID: 9569797
160. Sener M, Pektas ZO, Yilmaz I, Turkoz A, Uckan S, Donmez A, et al. Comparison of preemptive analgesic effects of a single dose of nonopioid analgesics for pain management after ambulatory surgery: a prospective, randomized, single-blind study in Turkish patients. Curr Ther Res Clin Exp. 2005;66(6):541-51. doi: 10.1016/j.curtheres.2005.12.001. PMID: 24678075
161. Sevekar SA, Gowda SHN. Postoperative pain and flare-ups: comparison of incidence between single and multiple visit pulpectomy in primary molars. J Clin Diagn Res. 2017;11(3):ZC09-ZC12. doi: 10.7860/JCDR/2017/22662.9377. PMID: 28511499
162. Seymour RA, Williams FM, Luyk NM, Boyle MA, Whitfield PM, Nicholson E, et al. Comparative efficacy of soluble aspirin and aspirin tablets in postoperative dental pain. Eur J Clin Pharmacol. 1986;30(4):495-8. PMID: 3743627
163. Seymour RA, Williams FM, Ward A, Rawlins MD. Aspirin metabolism and efficacy in postoperative dental pain. Br J Clin Pharmacol. 1984;17(6):697-701. doi: 10.1111/j.1365-2125.1984.tb02406.x. PMID: 6378231
164. Shah S, Shah SM. Dental trauma. Dtsch Arztebl Int. 2010;107(44):784-5. doi: 10.3238/arztebl.2010.0784c. PMID: 21116400
165. Sharma NK, Kindelan JD, Hutchinson D, Lancaster L. A study to compare ibuprofen effervescent granules with ibuprofen tablets in the treatment of acute dental pain. Prim Dent Care. 1994;1(1):5-8. PMID: 8941780
166. Sharpless J. Open wide. J Gen Intern Med. 2013;28(6):857-8. doi: 10.1007/s11606-012-2273-4. PMID: 23161354
167. Shimura K, Oto A, Hanai Y, Watanabe S, Toda M, Asada K, et al. Analgesic effect of fentiazac after tooth extraction or minor oral surgery. Clin Ther. 1981;4(1):12-7. PMID: 6974045
168. Siddique I, Mahmood H, Mohammed-Ali R. Paracetamol overdose secondary to dental pain: a case series. Br Dent J. 2015;219(6):E6. doi: 10.1038/sj.bdj.2015.706. PMID: 26405004
169. Silva de OJC, Grossi de OGA, Bassi AP. Comparative Assessment of the Effect of Ibuprofen and Etodolac on Edema, Trismus, and Pain in Lower Third Molar Surgery: a Randomized Clinical Trial. J Oral Maxillofac Surg. 2016;74(8):1524-30. doi: 10.1016/j.joms.2016.04.003. PMID: 27160363
170. Sindet-Pedersen S, Petersen JK, Gøtzsche PC, Christensen H. A doubleblind, randomized study of naproxen and acetylsalicylic acid after surgical removal of impacted lower third molars. Int J Oral Maxillofac Surg. 1986;15(4):389-94. PMID: 3091717
171. Sisk AL, Grover BJ. A comparison of preoperative and postoperative naproxen sodium for suppression of postoperative pain. Journal of oral and maxillofacial surgery. 1990;48:674-8.
172. Sisk AL, Mosley RO, Martin RP. Comparison of preoperative and postoperative diflunisal for suppression of postoperative pain. J Oral Maxillofac Surg. 1989;47(7):464-8. PMID: 2358942
173. Skjelbred P. The effects of acetylsalicylic acid on swelling, pain and other events after surgery. Br J Clin Pharmacol. 1984;17(4):379-84. doi: 10.1111/j.1365-2125.1984.tb02361.x. PMID: 6372841
174. Skoglund LA, Pettersen N. Effects of acetaminophen after bilateral oral surgery: double dose twice daily versus standard dose four times daily. Pharmacotherapy. 1991;11(5):370-5. PMID: 1745623
175. Skoglund LA, Skjelbred P. Comparison of a traditional paracetamol medication and a new paracetamol/paracetamol-methionine ester combination. Eur J Clin Pharmacol. 1984;26(5):573-7. PMID: 6468471
176. Skoglund LA. A new paracetamol/paracetamol-methionine ester combination effects on postoperative course. Eur J Clin Pharmacol. 1986;31(1):45-8. PMID: 3780826
177. Steffen P, Krinn E, Moller A, Seeling W, Rockemann MG. Metamizol and diclofenac profoundly reduce opioid consumption after minor trauma surgery. Acute pain. 2002;4(2):71-5. https://doi.org/10.1016/S1366-0071(02)00027-X
178. Sunshine A, Marrero I, Olson NZ, Laska EM, McCormick N. Oral analgesic efficacy of suprofen compared to aspirin, aspirin plus codeine, and placebo in patients with postoperative dental pain. Pharmacology. 1983;27(Suppl 1):31-40. doi: 10.1159/000137897. PMID: 6361789
179. Sunshine A, Olson NZ, Marrero I, Tirado S. Onset and duration of analgesia for low-dose ketoprofen in the treatment of postoperative dental pain. J Clin Pharmacol. 1998;38(12):1155-64. PMID: 11301569
180. Swift JQ, Garry MG, Roszkowski MT, Hargreaves KM. Effect of flurbiprofen on tissue levels of immunoreactive bradykinin and acute postoperative pain. J Oral Maxillofac Surg. 1993;51(2):112-6; discussion 116-7. PMID: 8426248
181. Symington JM, Listrom RD. A single dose study of a new analgesic. Ciramadol. J Can Dent Assoc. 1988;54(9):675-8. PMID: 3052729
182. Syrjanen SM, Syrjanen KJ. A new combination of drugs intended to be used as a preventive measure for the postextraction complications. A preliminary report. Int J Oral Surg. 1981;10(1):17-22. PMID: 6792091
183. Tai YM, Baker R. Comparison of controlled-release ketoprofen and diclofenac in the control of post-surgical dental pain. J R Soc Med. 1992;85(1):16-8. PMID: 1548648
184. Takarada H, Kinebuchi T, Kikuta T, Kondo T. Clinical effects of Napanol, a non-steroidal anti-inflammatory, anti-pyretic, analgesic agent for minor oral surgery and temporomandibular joint diseases. Shikai Tenbo. 1981;58(2):373-82. PMID: 6975500
185. Todd PA, Heel RC. Suprofen: A Review of Its Pharmacodynamic and Pharmacokinetic Properties, and Analgesic Efficacy. Drugs. 1985;30(6):514-38. doi: 10.2165/00003495-198530060-00004. PMID: 3908075
186. Tong SE, Daniels SE, Black P, Chang S, Protter A, Desjardins PJ. Novel p38α mitogen-activated protein kinase inhibitor shows analgesic efficacy in acute postsurgical dental pain. J Clin Pharmacol. 2012;52(5):717-28. doi: 10.1177/0091270011405496. PMID: 21659629
187. Trindade PA, Giglio FP, Colombini-Ishikiriama BL, Calvo AM, Modena KC, Ribeiro DA, et al. Sublingual ketorolac and sublingual piroxicam are equally effective for postoperative pain, trismus, and swelling management in lower third molar removal. Oral Surg Oral Med Oral Pathol Oral Radiol. 2012;114(1):27-34. doi: 10.1016/j.tripleo.2011.05.027. PMID: 22732846
188. Trindade PA, Giglio FP, Colombini-Ishikiriama BL, Calvo AM, Modena KC, Ribeiro DA, et al. Comparison of oral versus sublingual piroxicam during postoperative pain management after lower third molar extraction. Int J Oral Maxillofac Surg. 2011;40(3):292-7. doi: 10.1016/j.ijom.2010.10.026. PMID: 21144709
189. Troullos ES, Hargreaves KM, Butler DP, Dionne RA. Comparison of nonsteroidal anti-inflammatory drugs, ibuprofen and flurbiprofen, with methylprednisolone and placebo for acute pain, swelling, and trismus. J Oral Maxillofac Surg. 1990;48(9):945-52. PMID: 2395047
190. Tucci J, Bandiera E, Darwiche R, Medos Z, Nashed R, Trinh D. A review of the efficacy and safety of paracetamol and ibuprofen in the treatment of paediatric pain and fever. Aust J Pharm. 2009;90(1075):58-63.
191. Tuffin JR, Cunliffe DR, Begg R, Shaw SR. Does bupivacaine irrigation of third molar sockets reduce postoperative pain? A double blind controlled trial. Br J Oral Maxillofac Surg. 1990;28(2):96-8. PMID: 2186802
192. Turcotte JY. Ibuprofen pre- and postoperatively in oral surgery. J Can Dent Assoc. 1986;52(4):325-8. PMID: 3518886
193. Tuzuner OAM, Yazicioglu D, Alanoglu Z, Demiralp S, Ozturk A, Ucok C. Postoperative analgesia in impacted third molar surgery: the role of preoperative diclofenac sodium, paracetamol and lornoxicam. Med Princ Pract. 2011;20(5):470-6. doi: 10.1159/000327658. PMID: 21757939
194. Utsunomiya Y, Sotoike H, Shirosawa T, Kamata N, Omori N. Analgesic effect of Feldene (piroxicam) on pain following minor oral surgery. Shikai Tenbo. 1983;62(4):835-9. PMID: 6582633
195. Van Aken H, Thys L, Veekman L, Buerkle H. Assessing analgesia in single and repeated administrations of propacetamol for postoperative pain: comparison with morphine after dental surgery. Anesth Analg. 2004;98(1):159-65, table of contents. PMID: 14693612
196. Varner J, Lomax M, Blum D, Quessy S. A randomized, controlled, dose-ranging study investigating single doses of GW406381, naproxen sodium, or placebo in patients with acute pain after third molar tooth extraction. Clin J Pain. 2009;25(7):577-83. doi: 10.1097/AJP.0b013e3181a085fa. PMID: 19692798
197. Voelker M, Schachtel BP, Cooper SA, Gatoulis SC. Efficacy of disintegrating aspirin in two different models for acute mild-to-moderate pain: sore throat pain and dental pain. Inflammopharmacology. 2016;24(1):43-51. doi: 10.1007/s10787-015-0253-0. PMID: 26603742
198. Walton GM, Rood JP, Snowdon AT, Rickwood D. Ketorolac and diclofenac for postoperative pain relief following oral surgery. Br J Oral Maxillofac Surg. 1993;31(3):158-60. PMID: 8512909
199. Ward A, Brogden RN. Nimesulide: A Preliminary Review of its Pharmacological Properties and Therapeutic Efficacy in Inflammation and Pain States. Drugs. 1988;36(6):732-53. doi: 10.2165/00003495-198836060-00004. PMID: 3065059
200. Westhuijzen AJ, Roelofse JA, Grotepass FW, Becker PJ. Randomized double-blind comparison of tiaprofenic acid and diclophenac sodium after third molar surgery. Oral Surg Oral Med Oral Pathol. 1994;78(5):557-66. PMID: 7838460
201. White P, Strunin L. Post-anaesthetic dental extraction analgesia: a comparison of paracetamol, codeine, caffeine (Solpadeine) and diflunisal (Dolobid). Br J Oral Surg. 1982;20(4):275-80. PMID: 6961936
202. Williams JE, Ainley TC, Shepherd JE. Economic impact of a patientcontrolled oral analgesia system for post-operative pain. Br J Med Econ. 1995;9:41-3.
203. Winter L, Post A. Double-blind comparison of single oral doses of oxaprozin, aspirin, and placebo for relief of post-operative oral surgery pain. J Int Med Res. 1983;11(5):308-14. doi: 10.1177/030006058301100511. PMID: 6357891
204. Wright G, Smith A. Intra-muscular ketorolac administered as a supplemental analgesic for removal of impacted third molar teeth: a prospective study. Aust Dent J. 2002;47(1):41-4. PMID: 12035957
205. Yoshimasu H, Yoshikane K, Nakano Y, Iwata J, Yokoo E. An anti-inflammatory analgesic, Feldene (piroxicam), for pain following tooth extraction—clinical results. Shikai Tenbo. 1983;62(4):829-34. PMID: 6607545
206. Young CL, Strand V, Altman R, Daniels S. A phase 2 study of naproxen submicron particle capsules in patients with post-surgical dental pain. Adv Ther. 2013;30(10):885-96. doi: 10.1007/s12325-013-0057-9. PMID: 24127200
207. Yue Y, Collaku A, Brown J, Buchanan WL, Reed K, Cooper SA, et al. Efficacy and speed of onset of pain relief of fast-dissolving paracetamol on postsurgical dental pain: two randomized, single-dose, double-blind, placebo-controlled clinical studies. Clin Ther. 2013;35(9):1306-20. doi: 10.1016/j.clinthera.2013.07.422. PMID: 23972577
208. Yuki K, Shioiri S, Hashimoto K, Shimizu M, Shioda S. Use of a new anti-inflammatory analgesic, Froben granules, in oral surgery. Shikai Tenbo. 1980;55(1):169-73. PMID: 6931042
209. Zelenakas K, Fricke JR, Jayawardene S, Kellstein D. Analgesic efficacy of single oral doses of lumiracoxib and ibuprofen in patients with postoperative dental pain. Int J Clin Pract. 2004;58(3):251-6. PMID: 15117091
210. Ziccardi VB, Desjardins PJ, Daly-DeJoy E, Seng GF. Single-dose vicoprofen compared with acetaminophen with codeine and placebo in patients with acute postoperative pain after third molar extractions. J Oral Maxillofaci Surg. 2000;58(6):622-8. PMID: 10847283
211. Zor Z, Işik B, Çetiner S. Efficacy of pre-emptive lornoxicam on postoperative analgesia and edema after surgical removal of mandibular third molars. J Oral Maxillofac Surg. 2011;69(9):e-53. doi: https://doi.org/10.1016/j.joms.2011.06.101
212. Zor Z, Işik B, Çetiner S. Efficacy of preemptive lornoxicam on postoperative analgesia after surgical removal of mandibular third molars. Oral Surg Oral Med Oral Pathol Oral Radiol. 2014;117(1):27-31. doi: 10.1016/j.oooo.2013.08.027. PMID: 24332324
213. Zuniga JR, Malmström H, Noveck RJ, Campbell JH, Christensen S, Glickman RS, et al. Controlled phase III clinical trial of diclofenac potassium liquid-filled soft gelatin capsule for treatment of postoperative dental pain. J Oral Maxillofac Surg. 2010;68(11):2735-42. doi: 10.1016/j.joms.2010.05.075. PMID: 20869152
214. Zuniga JR, Noveck RJ, Schmidt WK, Boesing SE, Hersh EV. Onset of action of diclofenac potassium liquid-filled capsules in dental surgery patients. Curr Med Res Opin. 2011;27(9):1733-9. doi: 10.1185/03007995.2011.600300. PMID: 21770716
215. Zuniga JR, Phillips CL, Shugars D, Lyon JA, Peroutka SJ, Swarbrick J, et al. Analgesic safety and efficacy of diclofenac sodium softgels on postoperative third molar extraction pain. J Oral Maxillofac Surg. 2004;62(7):806-15. PMID: 15218558
216. Zupelari-Goncalves P, Weckwerth GM, Calvo AM, Simoneti LF, Dionisio TJ, Brozoski DT, et al. Efficacy of oral diclofenac with or without codeine for pain control after invasive bilateral third molar extractions. Int J Oral Maxillofac Surg. 2017;46(5):621-7. doi: 10.1016/j.ijom.2017.01.008. PMID: 28161136
