# Supplementary material for: Effects and cost-effectiveness of postoperative oral analgesics for additional postoperative pain relief in children and adolescents undergoing dental treatment: Health technology assessment including a systematic review
Source: PLoS One. 2019 Dec 31;14(12):e0227027. doi: 10.1371/journal.pone.0227027 (PMC6938383; doi:10.1371/journal.pone.0227027)
Supplement: Attachment — Submitted filename: Response to Reviewers.docx [file pone.0227027.s006.docx]

**Dear Academic Editor Federico Bilotta,**

Thank you for the positive response on our manuscript [PONE-D-19-18628] *Effects and cost-effectiveness of postoperative oral analgesics for additional postoperative pain relief in children and adolescents undergoing dental treatment: Health technology assessment including a systematic review*.

We have considered the Journal Requirements and comments and critique from the reviewers. We have made changes accordingly, but also have following comments:

**JOURNAL REQUIREMENTS:**

When submitting your revision, we need you to address these additional requirements.

1. Please ensure that your manuscript meets PLOS ONE's style requirements, including those for file naming. The PLOS ONE style templates can be found at <http://www.journals.plos.org/plosone/s/file?id=wjVg/PLOSOne_formatting_sample_main_body.pdf> and <http://www.journals.plos.org/plosone/s/file?id=ba62/PLOSOne_formatting_sample_title_authors_affiliations.pdf>

***[Authors’ comments]*: We have updated the file names according to the links above, and adjusted the manuscript to meet PLOS ONE’s style requirements.**

2. Thank you for stating the following in the Acknowledgments Section of your manuscript: "This study was supported by research funds from Oral Health Related Research by Region Skåne (Odontologisk Forskning i Region Skåne, OFRS 569491), Sweden". We note that you have provided funding information that is not currently declared in your Funding Statement. However, funding information should not appear in the Acknowledgments section or other areas of your manuscript. We will only publish funding information present in the Funding Statement section of the online submission form. Please remove any funding-related text from the manuscript and let us know how you would like to update your Funding Statement. Currently, your Funding Statement reads as follows:

"The funders had no role in study design, data collection and analysis, decision to publish, or preparation of the manuscript".

***[Authors’ comments]*: The funding information is removed from the manuscript. For the Funding section, we would like to update the text with the following:**

*“This study was supported by research funds from Oral Health Related Research by Region Skåne (Odontologisk Forskning i Region Skåne, OFRS 569491), Sweden.*

*The funders had no role in study design, data collection and analysis, decision to publish, or preparation of the manuscript.”*

3. Please include a copy of Table 1 which you refer to in your text on page 6.

***[Authors’ comments]***: **Table 1 is now included in present re-submission.**

**REVIEWERS’ COMMENTS:**

Reviewer's Responses to Questions

Comments to the Author

1. Is the manuscript technically sound, and do the data support the conclusions?

The manuscript must describe a technically sound piece of scientific research with data that supports the conclusions. Experiments must have been conducted rigorously, with appropriate controls, replication, and sample sizes. The conclusions must be drawn appropriately based on the data presented.

Reviewer #1: No

***[Authors’ comments]*:** **The manuscript has been prepared according to common principles for systematic reviews (SR) and health technology assessments (HTA). We have followed the guidelines for SRs, from SBU (Swedish Agency for Health Technology Assessment and Assessment of Social Services) (Line 393-397, page 16). SBU is in the same network as Cochrane Sweden, which is a part of the Cochrane global community. This SR also follows the PRISMA-statement (Line 158-159, page 6)**

Reviewer #2: Yes

2. Has the statistical analysis been performed appropriately and rigorously?

Reviewer #1: No

***[Authors’ comments]*:** **Since this SR came out empty, no statistical analysis could be performed. This question is to our understanding therefore not applicable.**

Reviewer #2: N/A

3. Have the authors made all data underlying the findings in their manuscript fully available?

The PLOS Data policy requires authors to make all data underlying the findings described in their manuscript fully available without restriction, with rare exception (please refer to the Data Availability Statement in the manuscript PDF file). The data should be provided as part of the manuscript or its supporting information, or deposited to a public repository. For example, in addition to summary statistics, the data points behind means, medians and variance measures should be available. If there are restrictions on publicly sharing data—e.g. participant privacy or use of data from a third party—those must be specified.

Reviewer #1: No

***[Authors’ comments]*: Reviewer #1 has not given any thorough review on what is missing. We therefore find it difficult to revise the manuscript, without this detailed information.**

Reviewer #2: Yes

4. Is the manuscript presented in an intelligible fashion and written in standard English?

PLOS ONE does not copyedit accepted manuscripts, so the language in submitted articles must be clear, correct, and unambiguous. Any typographical or grammatical errors should be corrected at revision, so please note any specific errors here.

Reviewer #1: No

***[Authors’ comments]*: Language editing was undertaken before submitting the manuscript. This was done by an English speaking professional English Language Consultant with long experience in scientific language.**

Reviewer #2: Yes

5. Review Comments to the Author

Reviewer #1:

• The preparation of Systematic Review was erroneous. Several lacunae were noted and authors should discuss with someone who has prepared SRs before, especially those who have worked with the Cochrane Collaboration.

***[Authors’ comments]*: This systematic review has been performed according to the guidelines from the Swedish Agency for Health Technology Assessment and Assessment of Social Services (SBU), which is a Swedish governmental agency focusing on assessing and evaluating methods in use in healthcare and social services. It is one of the world’s oldest HTA agencies, collaborating with Cochrane Sweden. We have referred to this in the manuscript (Line 175-178, side 7-8; ref #16** *(Swedish Agency for Health Technology Assessment and Assessment of Social Services (SBU). Assessment of methods in health care—A handbook. Stockholm. 2018. Available:* [*https://www.sbu.se/contentassets/76adf07e270c48efaf67e3b560b7c59c/eng_metodboken.pdf)*](https://www.sbu.se/contentassets/76adf07e270c48efaf67e3b560b7c59c/eng_metodboken.pdf))**). The PRISMA-statement has been followed as well (Line 159, side 6; ref #14** *(Moher D, Liberati A, Tetzlaff J, Altman DG, The PG. Preferred reporting items for systematic reviews and meta-analyses: the PRISMA statement. PLoS Med. 2009;6(7):e1000097. doi: 10.1371/journal.pmed.1000097. PMID: 19621072)***).**

• Outcomes are not well defined and the PICO statement is incorrectly prepared.

***[Authors’ comments]*: We believe that the PICO is formulated correctly. We have defined the Population (Line 104, page 4), Interventions (Line 108, page 4 and Line 109-114, page 5), Control (line 116-124, page 5), and Outcome Measures (line 126-130, page 5). The SR has also been registered on PROSPERO (**[**https://www.crd.york.ac.uk/PROSPERO/**](https://www.crd.york.ac.uk/PROSPERO/)**).**

• Professional help should have been taken for preparation of Search Strategies.

***[Authors’ comments]*: This has been made (line 155-156, page 6). Two librarians specialized in informatics performed the search in all five databases, used in this SR.**

Reviewer #2 (only the bullet points requiring revision):

• Please, add a PRISMA statement checklist.

***[Authors’ comments]*: PRISMA checklist was added in the first submission. We have however added it again with the current re-submission**.

• Please, add an AMSTAR checklist.

***[Authors’ comments]*: This is added with the current re-submission.**

• Please, define Limitations of the study.

***[Authors’ comments]*: Limitations of our study are mentioned, see Line 268-286, page 11-12. However we have inserted a phrase in the beginning of Line 268, page 11, and at the end of the first sentence on line 275, page 11, to make it clearer that limitations are discussed. “*Possible limitations of this SR could be that the outcome measure (pain after dental treatment assessed by the child patient) was too narrow*.”, and “*The definition of the population could also be discussed, as a limitation of this SR*.”**

Thank you for your valuable comments and critique! We hope you will find the changes and clarifications satisfactory. We look forward to hearing from you regarding our re-submission. We would be glad to respond to any further questions and comments that you may have.

Yours sincerely,

Henrik Berlin,

Main Author, DDS

Correspondence address:	Henrik Berlin

Department of Pediatric Dentistry

Faculty of Odontology

Malmö University

SE-205 06 Malmö, Sweden
